# Brain Tumor Segmentation and Grading on MRI Using Deep Learning: A Systematic Literature Review and Benchmark-Driven Comparative Analysis

**DOI:** 10.3390/diagnostics16172806

**Published:** 2026-08-31

**Authors:** Lama Almudaimeegh, Kholoud Alwashmi, Zuhal Y. Hamd

**Affiliations:** 1 Department of Internal Medicine, College of Medicine, Princess Nourah bint Abdulrahman University, P.O. Box 84428, Riyadh 11671, Saudi Arabia; l.almudaimeegh@pnu.edu.sa; 2 Department of Radiological Sciences, College of Health and Rehabilitation Sciences, Princess Nourah bint Abdulrahman University, P.O. Box 84428, Riyadh 11671, Saudi Arabia; zyhamd@pnu.edu.sa

**Keywords:** brain tumor segmentation, glioma grading, deep learning, magnetic resonance imaging, U-Net, vision transformer, BraTS challenge, systematic literature review, explainable artificial intelligence, reproducibility

## Abstract

**Background**: Magnetic resonance imaging (MRI) is central to brain tumor segmentation and histological grading, and deep learning (DL) has transformed both tasks. Existing reviews rarely span the 2017–2026 architectural arc from CNNs and U-Net variants to transformers and foundation models or appraise reproducibility and clinical-translation readiness. **Methods**: This preregistered systematic review (PRISMA 2020, PRISMA-S) searched Google Scholar, PubMed/MEDLINE, and IEEE Xplore on 21 May 2026 (January 2017–May 2026). A single reviewer performed screening, extraction and QUADAS-AI appraisal with repeated checks on separate days; therefore, the synthesis is presented as a transparent descriptive review rather than a pooled meta-analysis. Records were screened against a priori eligibility criteria; primary experimental studies entered the synthesis and review articles formed a contextual corpus. Methodological quality was appraised using an adapted QUADAS framework (“QUADAS-AI”). Heterogeneity precluded statistical pooling, so a benchmark-driven comparative synthesis was conducted. **Results**: The search retrieved 33,982 records (Google Scholar 23,400; PubMed/MEDLINE 5349; IEEE Xplore 5233). After deduplication and screening, 141 full texts were assessed; one report published before the eligibility window was excluded, leaving 140 included studies: 117 primary (39 contributed to the BraTS Dice benchmark sub-set) and 23 contextual reviews. U-Net variants (42%) and hybrid CNN–transformer architectures (40%) dominate, followed by CNN classifiers (14%) and vision transformers (3%). On BraTS 2021, nnU-Net and Swin UNETR reach DSC 0.93/0.90/0.85 (whole tumor/core/enhancing); reported external evaluations show 2.5–15 percentage-point performance drops under domain shift. **Conclusions**: External generalizability, uncertainty quantification, reproducibility, and prospective validation remain weak; ten research priorities are proposed. **Registration**: OSF, DOI 10.17605/OSF.IO/C2QA6; registered 20 May 2026.

## 1. Background

Primary brain and central nervous system (CNS) tumors represent a heterogeneous group of neoplasms with widely varying prognoses. The Central Brain Tumor Registry of the United States (CBTRUS) estimates that 107,100 new malignant and non-malignant primary brain and other CNS tumors were expected to be diagnosed in the United States in 2025, with an overall five-year relative survival rate of 34.8% for malignant histologies [1]. Glioblastoma (GBM), the most aggressive grade IV glioma, carries a median overall survival of approximately 15 months under maximal safe resection followed by concurrent chemo-radiation with temozolomide [2]. The 2021 World Health Organization (WHO) Classification of CNS Tumors introduced molecular markers—isocitrate dehydrogenase (IDH) mutation status, 1p/19q co-deletion, and TERT promoter mutations—as integral diagnostic criteria that supersede histological grading in certain contexts [3].

Multi-parametric MRI (mpMRI) comprising T1-weighted (T1), T1 contrast-enhanced (T1ce), T2-weighted (T2), and T2 fluid-attenuated inversion recovery (T2-FLAIR) sequences is the non-invasive modality of choice for brain tumor diagnosis, treatment planning, and postoperative surveillance. Manual delineation of tumor sub-regions—whole tumor (WT), tumor core (TC), and enhancing tumor (ET)—is labor-intensive (30–60 min per case), subjective, and exhibits high inter-rater variability with Dice overlap as low as 0.74–0.85 between experienced neuroradiologists [4]. This bottleneck has motivated intensive research into automated segmentation and computer-aided grading using deep learning (DL).

The seminal U-Net architecture [5] established the encoder–decoder paradigm with skip connections that remains foundational to medical image segmentation. Subsequent advances include the self-configuring nnU-Net [6], transformer-based models such as TransBTS [7], UNETR [8], and Swin UNETR [9], as well as emerging state-space models (SSM) including U-Mamba [10] and SegMamba [11]. The BraTS challenge series, running annually since 2012, has served as the primary benchmark for evaluating these methods on standardized, multi-institutional datasets [4,12,13].

Despite the proliferation of primary research, several gaps motivate this review. First, existing surveys typically address either segmentation or classification in isolation [14,15,16]. Second, the rapid emergence of transformers, hybrid architectures, and Mamba-based models has outpaced most published reviews. Third, BraTS expanded from a single adult-glioma task to eight sub-challenges across four tumor types and diverse geographies from 2023–2024, yet no review aggregates results across all tracks [17,18]. Fourth, few reviews critically examine external validation, domain shift, and uncertainty quantification.

This systematic literature review addresses these gaps by: (a) covering both segmentation and grading within a unified PRISMA-compliant methodology; (b) spanning nine years of research (2017–2026); (c) performing a benchmark-driven comparative analysis across multiple BraTS tracks; (d) providing a taxonomic classification of six architectural families; (e) conducting formal quality assessment using an AI-adapted QUADAS-AI framework with explicit reproducibility and clinical-translation-readiness domains; and (f) identifying concrete, actionable future research directions grounded in empirical evidence.

## 2. Methods

This review was conducted in accordance with the PRISMA 2020 statement [19]. The workflow comprised protocol registration, database searching, deduplication, title/abstract screening, full-text eligibility assessment, structured data extraction, QUADAS-AI appraisal, and descriptive benchmark-driven synthesis. “Systematic review” and “systematic literature review” (SLR) are used as equivalent terms throughout.

### 2.1. Protocol and Registration

The protocol for this systematic literature review (SLR) was preregistered prospectively on the Open Science Framework (OSF) prior to commencement of the formal search and screening process. The registration is titled Brain Tumor Segmentation and Grading on MRI Using Deep Learning: A Systematic Literature Review and Benchmark-Driven Comparative Analysis (DOI 10.17605/OSF.IO/C2QA6, https://osf.io/c2qa6 (accessed on 30 July 2026)); it was registered on 20 May 2026, and the final database search was executed on 21 May 2026. Reporting follows PRISMA 2020 [19] and the PRISMA-S extension for search-strategy reporting. The completed PRISMA 2020 checklist is provided in Supplementary File S1, while the database-specific search strategies, filters, search dates, hit counts, and stopping rules are provided in Supplementary File S2. Preliminary scoping informed protocol development; the formal database search and eligibility screening were conducted after OSF registration.

### 2.2. Research Questions

This SLR is guided by five research questions (RQs):

RQ1: What DL architectural families have been proposed for brain tumor segmentation and grading on MRI, and how has the landscape evolved from 2017 to 2026?

RQ2: What are the state-of-the-art quantitative performances on standardized benchmarks (BraTS editions, Figshare, Kaggle), and how do they compare across architecture types?

RQ3: What are the most common loss functions, optimizers, preprocessing pipelines, and data-augmentation strategies employed, and how do they influence outcomes?

RQ4: To what extent do current models generalize across institutions, MRI protocols, and patient populations, and what are the main sources of performance degradation?

RQ5: What are the critical open challenges and actionable future research directions for clinical translation of DL-based brain tumor analysis?

### 2.3. Eligibility Criteria

Consistent with the OSF preregistration, primary experimental studies and contextual review articles were treated as two distinct corpora:

- Primary experimental studies were eligible for the systematic synthesis and the benchmark-driven comparative analysis.
- Review articles (systematic reviews, narrative reviews, surveys, meta-analyses by other authors) were not included in the quantitative benchmark synthesis; they were retained as a contextual corpus used only for background discussion, citation tracking, and comparison with prior reviews comparison with prior reviews, as discussed in Section 4.2.

Inclusion criteria (primary studies).

- Peer-reviewed journal articles, conference papers, or eligible preprints published between January 2017 and the final search date (21 May 2026), inclusive of records identified through the presubmission search update.
- Studies proposing or evaluating deep learning models for brain tumor segmentation, classification, grading, or detection from MRI data.
- Quantitative evaluation using at least one standard metric (DSC, Hausdorff distance/HD95, accuracy, sensitivity, specificity, precision, F1-score, AUC, IoU).
- Evaluation on benchmark datasets (any BraTS edition, Figshare, Kaggle Brain MRI, TCGA, REMBRANDT, Br35H) or institutional datasets with sufficient sample size as reported in the study.

Exclusion criteria.

- Non-MRI modalities (CT, PET, ultrasound) without an MRI component.
- Pure radiomics or hand-crafted-feature methods without a deep learning component.
- Non-English publications.
- Editorials, opinion pieces, or abstracts without original experimental results.
- Studies evaluating only overall survival prediction without segmentation, classification, grading, or detection.
- Duplicate publications reporting the same experimental results (the most complete or peer-reviewed version was retained).
- Extremely small datasets with insufficient quantitative evaluation, as judged by the reviewer.

### 2.4. Information Sources and Search Strategy

Three electronic databases, registered in the OSF protocol, were searched on 21 May 2026: Google Scholar, PubMed/MEDLINE, and IEEE Xplore. No other databases (e.g., Scopus, Web of Science, Embase) were used. The conceptual query combined four concept blocks using AND operators:

(“brain tumor” OR “brain tumour” OR glioma OR glioblastoma OR meningioma OR astrocytoma) AND (segmentation OR classification OR grading OR detection) AND (MRI OR “magnetic resonance imaging” OR “magnetic resonance”) AND (“deep learning” OR “convolutional neural network” OR CNN OR U-Net OR transformer OR “neural network”)

The conceptual query was operationalized in database-specific syntax (title/abstract and MeSH for PubMed/MEDLINE, platform syntax for IEEE Xplore, simplified syntax for Google Scholar). The exact database-specific search strings, filters, date searched, raw hit counts, sort order, screening depth, and stopping rules are provided in Supplementary File S2 (PRISMA-S search-strategy report) and summarized in Table 1.

For Google Scholar, the relevance-based stopping rule (first 50 result pages, approximately 10 records per page) was adopted because Google Scholar lacks reproducible exhaustive export and result relevance declines rapidly beyond the first relevance-ranked pages, consistent with PRISMA-S guidance for Google Scholar as a supplementary source. The IEEE Xplore corpus retrieved under the conceptual query comprised 4655 conference papers, 507 journal articles, 35 early-access articles, 33 books and 3 magazine articles. Backward and forward citation tracking was performed on the included primary studies and on key contextual reviews. A presubmission search update was additionally conducted after OSF registration but before manuscript submission to capture newly available eligible studies; these records were screened against the same eligibility criteria and are flagged in the master data extraction sheet (Supplementary File S3).

### 2.5. Study Selection

**Figure 1:**
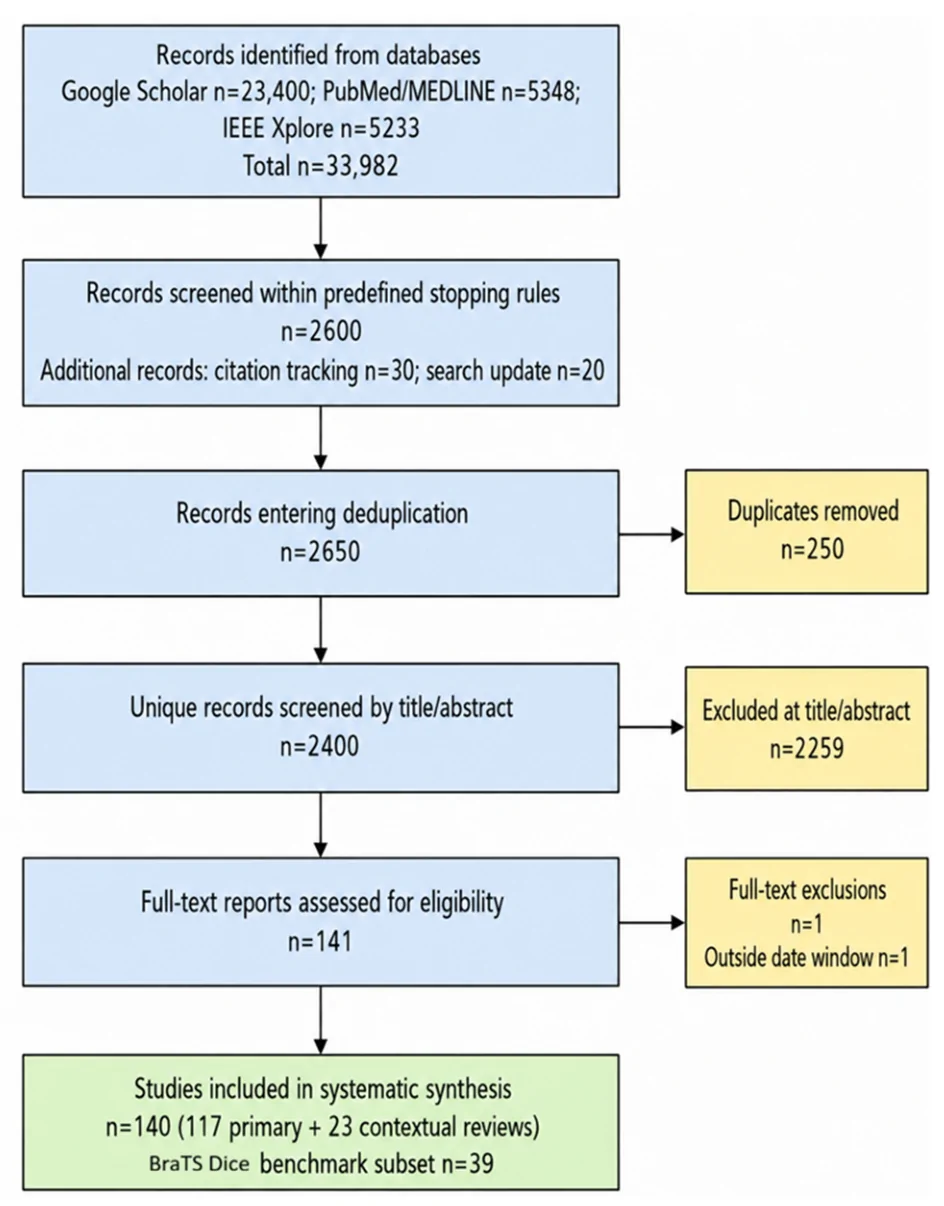
PRISMA 2020 flow diagram illustrating the study selection process. The database search retrieved 33,982 records; within predefined relevance-based stopping rules 2600 records were screened, supplemented by 50 records from citation tracking and a presubmission search update. After deduplication, two-stage screening and assessment of 141 full-text reports, one report outside the prespecified publication window was excluded and 140 studies were included in the systematic synthesis (117 primary studies + 23 contextual reviews), of which 39 primary studies reported sufficient Dice metrics on BraTS benchmarks to enter the benchmark-driven comparative analysis (Blue boxes denote records retained at each stage, yellow boxes denote removed or excluded records, and the green box denotes the final included corpus.).

The sole reviewer (ZYH) screened titles and abstracts against the predefined inclusion and exclusion criteria. Full-text articles were evaluated by the same reviewer, with each eligibility decision repeated on a separate day. Title/abstract decisions were likewise repeated on a separate day, and any inconsistency was resolved by re-reading the relevant record or full text. The complete selection workflow is depicted in the PRISMA 2020 flow diagram (Figure 1).

### 2.6. Data Extraction

For each included primary study, the following data were extracted into a structured spreadsheet (Supplementary File S3): (1) bibliographic information (authors, year, journal/venue, DOI, country of corresponding author, publication type); (2) clinical and task fields (tumor type, primary task, clinical target, type of validation); (3) imaging fields (MRI sequences, 2D/3D input, single vs. multi-sequence, scanner/field strength, preprocessing pipeline); (4) dataset fields (dataset name and edition, number of patients/images, train/validation/test split, internal vs. external test, public/private status, annotation method); (5) model fields (architecture family, exact model name, backbone, attention/transformer/Mamba components, ensemble, foundation-model use, federated/XAI/uncertainty/lightweight flags); (6) training fields (optimizer, loss function, learning rate, batch size, epochs, augmentation, hardware, parameters/FLOPs/runtime/memory when reported, ablation study); (7) evaluation fields (DSC WT/TC/ET, HD95, sensitivity, specificity, accuracy, precision, recall, F1, AUC, IoU, mAP, calibration, statistical comparisons, external validation); (8) reproducibility fields (code availability, dataset availability, pretrained model availability, hyperparameter completeness, limitations disclosed); (9) clinical-translation fields (explainability method, uncertainty quantification, reader study, prospective/multi-site validation, regulatory/deployment claims, automation-bias discussion); and (10) reviewer-critical comments (main strength, main limitation, possible metric inflation, dataset-leakage risk, train/test split clarity). Missing information was recorded as not reported. The complete extraction sheet (Supplementary File S3), contextual-reviews list (Supplementary File S5), and evidence-locked study decision log (Supplementary File S6) accompany this manuscript.

### 2.7. Quality Assessment

Methodological quality and risk of bias were appraised using a QUADAS-AI framework [20] adapted from QUADAS-2 for deep learning medical-imaging studies (Supplementary File S4), with seven domains:

1. Patient/Data selection: dataset curation, class-imbalance handling, justification of inclusion/exclusion of cases.
2. Reference standard/annotation quality: number of annotators, inter-rater agreement, STAPLE/consensus protocols, expert qualifications.
3. Index test/model development: model description completeness, hyperparameter reporting, ablation, training/validation/test splitting strategy.
4. Flow and timing/data-leakage risk: patient-level vs. slice-level splitting, retention of all enrolled cases, consistency between training and evaluation protocols.
5. External validation and generalizability: use of independent test sets, multi-site validation, domain-shift assessment.
6. Reproducibility and transparency: code availability, dataset availability, pretrained model availability, completeness of hyperparameters and seeds.
7. Clinical-translation readiness: explainability, uncertainty quantification, reader study, prospective evaluation, regulatory or deployment discussion.

**Figure 2:**
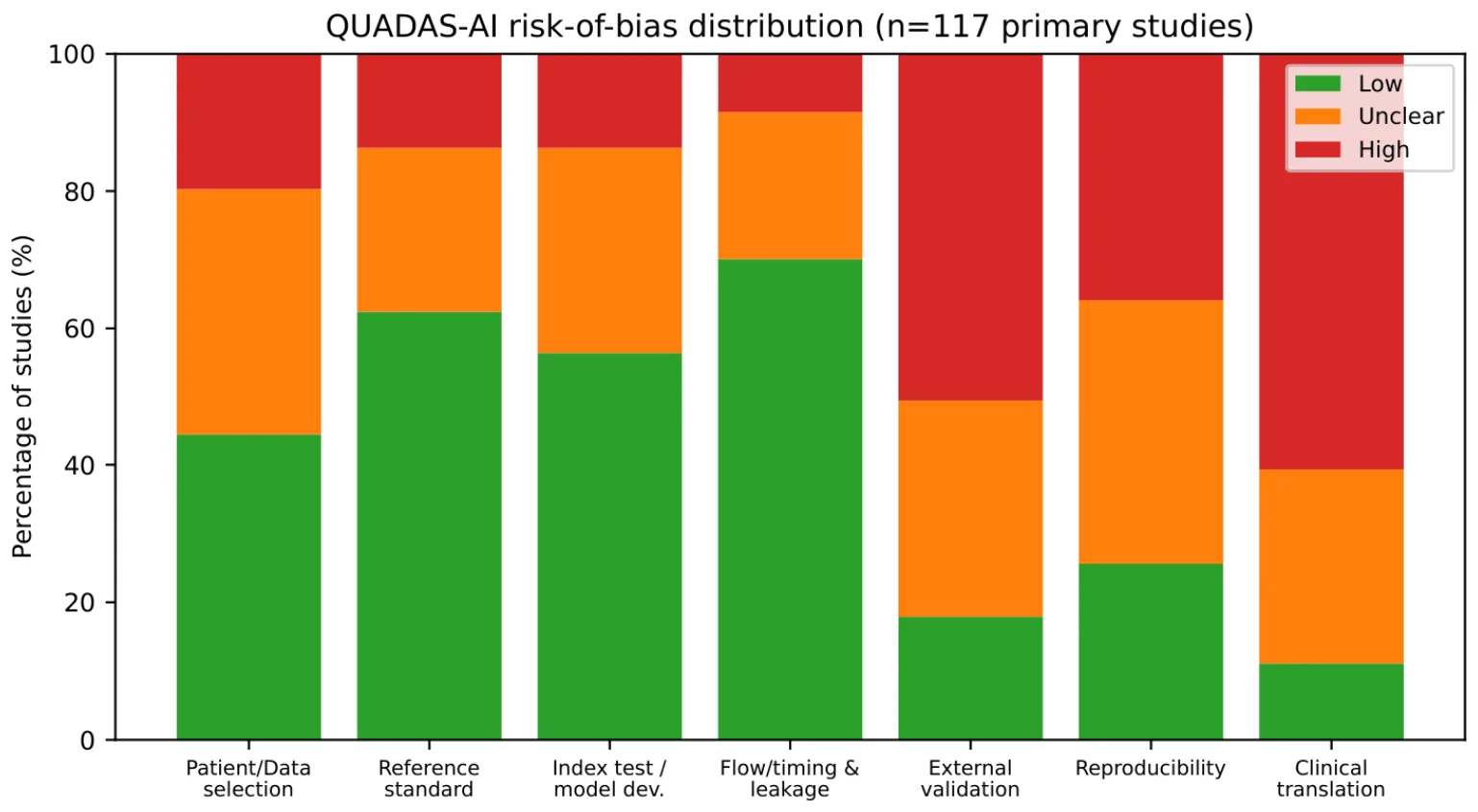
Quality assessment summary using the adapted QUADAS-AI framework applied to the 117 included primary studies. Stacked bars indicate the percentage of studies rated as low, unclear, or high risk of bias per domain; ratings are used to inform interpretation rather than to exclude studies.

Each domain was rated as low, unclear, or high risk of bias. The domain-level distribution, appraisal criteria, and interpretation summary are provided in Supplementary File S4 and summarized in Figure 2. Consistent with the preregistered protocol, studies were not excluded on the basis of quality scoring; quality ratings inform the narrative interpretation and a sensitivity discussion in Section 3.16 but do not alter the primary inclusion decision.

For transparency, the final QUADAS-AI domain counts were as follows for low/unclear/high risk, respectively, out of 117 primary studies: patient/data selection 52/42/23; reference standard/annotation quality 73/28/16; index test/model development 66/35/16; flow/timing/data-leakage risk 82/25/10; external validation/generalizability 21/37/59; reproducibility/transparency 30/45/42; and clinical-translation readiness 13/33/71. These counts highlight that the strongest recurring weaknesses are external validation, reproducibility, and clinical-translation readiness.

### 2.8. Data Synthesis Approach

Consistent with the preregistered analysis plan, no formal statistical meta-analysis was undertaken. Substantial heterogeneity across BraTS editions, evaluation protocols, dataset splits, and metric definitions precluded homogeneous statistical pooling; reported metrics across the corpus are therefore best interpreted descriptively. The synthesis was structured as follows:

- Segmentation: DSC (WT/TC/ET) and HD95 were tabulated per architecture family and per BraTS edition; best-reported values were extracted from each study without statistical pooling.
- Classification, grading and molecular prediction: accuracy, sensitivity, specificity, F1 and AUC were tabulated per task and per dataset.
- Structured comparative synthesis: results were stratified by architecture family, benchmark dataset, publication year, single-center vs. multi-center design, and internal vs. external validation.
- Descriptive subgroup comparison: where appropriate, distributions of reported metrics across subgroups were summarized using medians and interquartile ranges.
- Visualization: bar charts, heatmaps, distribution plots, radar charts and trend plots were generated from the extraction sheet using reproducible Python (version 3.13.5) scripts (scripts/generate_figures_reproducible.py).

Terminology such as pooled estimate, random-effects model, I^2^, forest plot, and meta-analysis is reserved for descriptions of other published reviews and is not applied to the present synthesis.

### 2.9. Reporting Bias

Formal funnel-plot asymmetry analysis and random-effects-based reporting-bias tests were not undertaken because they presuppose comparable effect sizes and homogeneous outcome definitions that are not satisfied by the heterogeneous benchmarks used in this literature. Risks of reporting bias are instead discussed narratively in Section 4.3.2, with explicit attention to potential over-representation of high-performing results, selective reporting of the best DSC subset or epoch, the predominance of single-dataset evaluations, and the use of Google Scholar as a supplementary source whose result ordering is not reproducible across queries. Study-selection decisions and the quantitative values reported in the benchmark tables (the quantitative values reported in the subsequent benchmark tables presented in Section 3.5 and Section 3.7) were checked against the available source records; eligibility and classification status are recorded in the unique-record decision log provided as Supplementary File S6.

## 3. Results

### 3.1. Study Selection and PRISMA Flow

The database search executed on 21 May 2026 retrieved 33,982 records: Google Scholar (23,400), PubMed/MEDLINE (5349), and IEEE Xplore (5233). Within the predefined relevance-based stopping rules (Section 2.4; Table 1), 2600 records were screened from these databases (Google Scholar approximately 500, PubMed/MEDLINE approximately 1100, IEEE Xplore approximately 1000). A further 50 records were identified through backward and forward citation tracking and the presubmission search update, giving 2650 records entering the deduplication step.

After removing 250 duplicates, 2400 unique records underwent title–abstract screening, from which 2259 were excluded for not meeting eligibility criteria (the database-specific search and screening details are reported in Supplementary File S2). Of 141 full-text articles assessed, one 2015 report was excluded because it fell outside the prespecified January 2017–21 May 2026 publication window. The complete full-text eligibility and classification decision log is provided in Supplementary File S6.

The final corpus comprises 140 studies: 117 primary experimental studies (entering the systematic synthesis) and 23 contextual review articles (used only as background, with a cross-reference summary provided in Section 4.2). Of the 117 primary studies, 39 reported a usable Dice similarity coefficient on a BraTS benchmark and form the quantitative benchmark subset that drives the comparative analysis (Section 3.5 onwards). The complete PRISMA 2020 flow is shown in Figure 1.

### 3.2. Temporal and Geographic Characteristics

**Figure 3:**
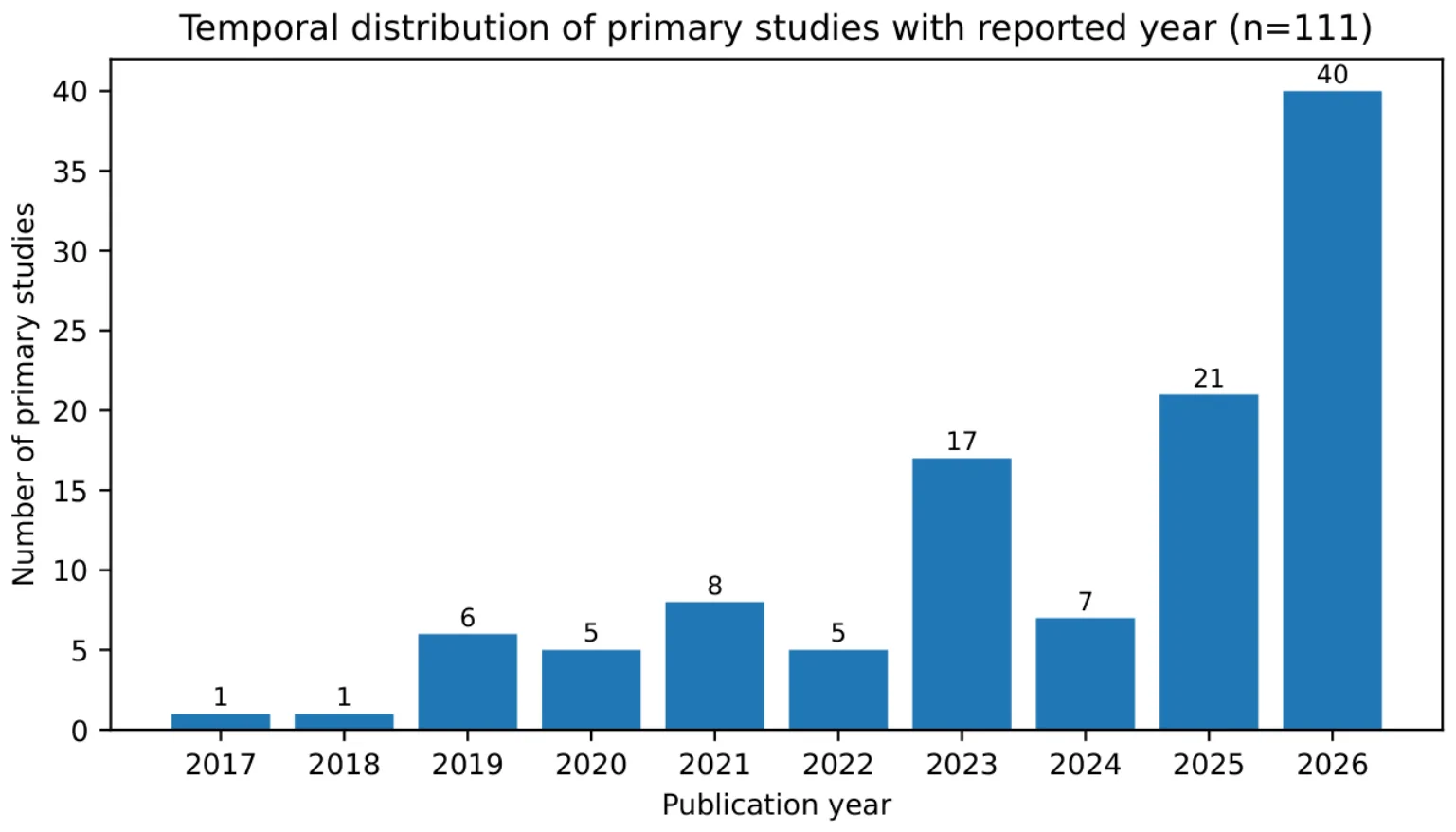
Temporal distribution of primary studies with a reported eligible publication year. The steep increase from 2023 onward reflects growing maturity and expanding clinical interest in DL-based brain tumor analysis on MRI.

The included corpus spans 2017–2026 with a marked acceleration in publication rate (Figure 3). Of the 111 primary studies with an extractable publication year between 2017 and 2026, the temporal distribution reveals five phases: two studies (2017–2018) established foundational CNN approaches; 11 (2019–2020) introduced encoder–decoder maturation with attention mechanisms; 13 (2021–2022) marked the transformer revolution; 24 (2023–2024) brought hybrid models and expanded benchmarks; and 61 studies (2025–May 2026) reflect the current era of emerging paradigms.

Studies originated from 28 countries. The most represented were China (24%), United States (16%), India (15%), South Korea (7%), Germany (6%), and Saudi Arabia (4%). Among the 140 included studies, 117 (84%) were primary experimental studies on MRI-based brain tumor segmentation, classification, grading, detection, molecular prediction, or multi-task analysis using deep learning, and the remaining 23 (16%) were systematic or narrative review articles included for contextual analysis. Task coverage across the 117 primary studies was heterogeneous rather than segmentation-universal. The quantitative analyses were therefore organized into task-specific, non-mutually-exclusive subsets: 39 primary studies reported usable Dice outcomes on BraTS benchmarks for the segmentation comparison, while 28 studies contributed classification results to the classification-focused quantitative synthesis. Additional primary studies addressed grading, detection, molecular prediction, or multi-task pipelines, as detailed in the structured study summary in Section 3.17. Because a multi-task study can contribute to more than one task category, task-specific subset counts are reported separately rather than as percentages that sum to 117.

### 3.3. Taxonomy of Deep Learning Architectures

We organize the included methods into six architectural families:

Family 1: Encoder–decoder CNNs (U-Net family), 42% of primary studies. This family encompasses the original U-Net [5], 3D U-Net [21], V-Net [22], Attention U-Net [23], and the self-configuring nnU-Net [6]. Recent variants include residual U-Net with channel and spatial attention (CBAM), 3D AGRes-UNet combining atrous spatial pyramid pooling (ASPP) with attention gates, RP-SegNet with recursive residual connections, DeepSeg with FLAIR-focused multi-scale processing [24], and the teacher–student framework for modality distillation.

Family 2: Deep CNN classifiers, 14% of primary studies. These employ backbone architectures such as ResNet [25], VGG [26], DenseNet, EfficientNet, and MobileNet for tumor-type classification and WHO grading [27,28,29]. Object-detection models (YOLOv7, YOLOv8, YOLOv12) have also been adapted for tumor localization [30,31].

Family 3: Vision transformers, 3% of primary studies. Key methods include TransBTS [7], UNETR [8], and Swin UNETR [9], which leverage multi-head self-attention to capture long-range spatial dependencies.

Family 4: Hybrid CNN–transformer models, 40% of primary studies. Architectures such as CoHeD-BrainNet, Bayesian DeepLabV3+, CT-UNet, MedNeXt [32], and SwitchNet combine the local feature extraction of convolutions with the global context modeling of transformers.

Family 5: Generative and augmentation models (used as auxiliary components within the above families). GANs and denoising diffusion probabilistic models (DDPMs) are used for synthetic data augmentation to address class imbalance and limited training data [33].

Family 6: Emerging paradigms (Mamba state-space models, foundation models, knowledge-distilled lightweight networks; reported within the hybrid CNN–transformer counts above when applicable). This category includes state-space models (U-Mamba [10], SegMamba [11]), a federated-learning framework for privacy-preserving multi-institutional training (subsequently retracted) [34], foundation-model fine-tuning approaches [29], and prompt-based segmentation models. Notably, the Segment Anything Model (SAM) [35] and its medical adaptations MedSAM [36] have demonstrated the potential of foundation-model prompting for medical image segmentation, achieving competitive brain tumor results with minimal task-specific fine-tuning and representing a paradigm shift toward interactive, zero-shot segmentation. In this review, foundation-model evidence is interpreted cautiously because most studies remain early-stage and require task-specific validation on volumetric MRI, scanner-diverse cohorts, and clinically realistic annotation workflows.

### 3.4. Architecture and Dataset Distribution

**Figure 4:**
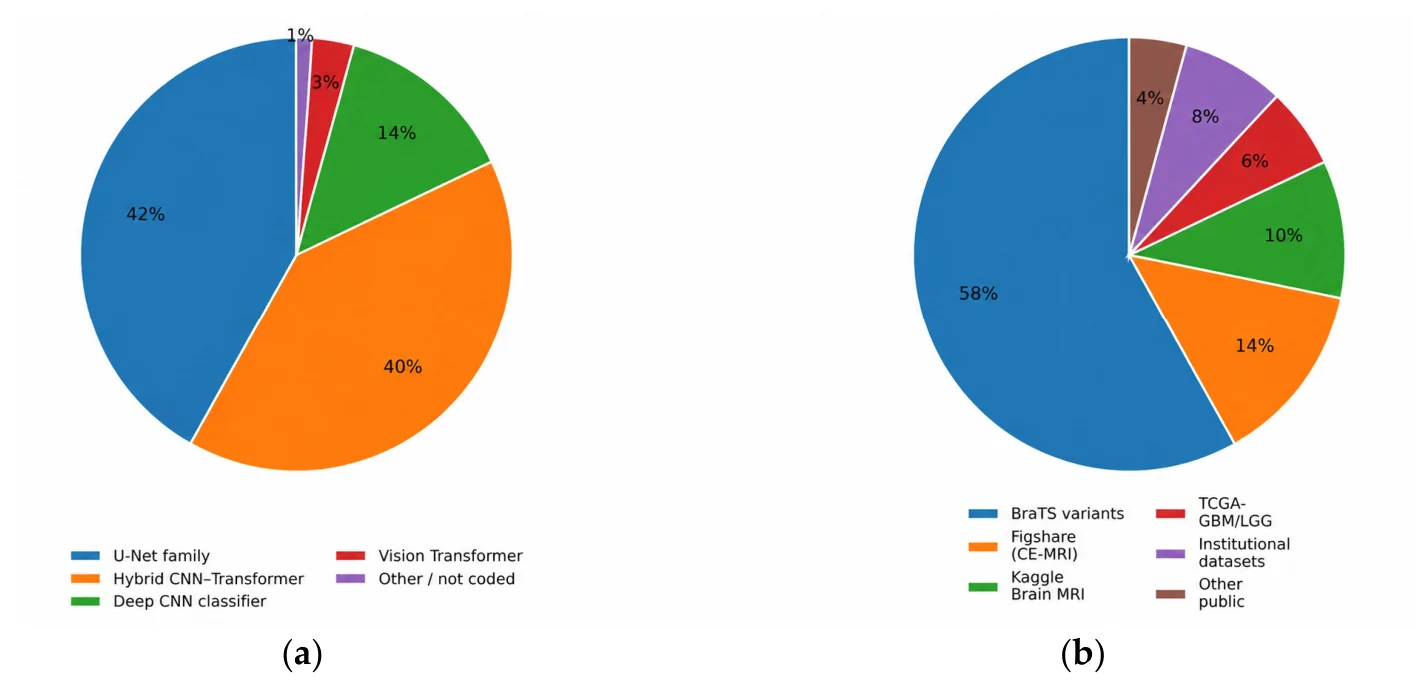
Distribution of (**a**) DL architecture families across the 117 primary studies and (**b**) benchmark dataset categories in the coded analytical corpus. BraTS variants dominate segmentation evaluations, while classification studies use more diverse but smaller-scale datasets.

Figure 4 illustrates the distribution of architectural families across the 117 primary studies and the dataset categories used in the coded analytical corpus. BraTS variants collectively constitute 58% of all datasets used, followed by Figshare (14%), Kaggle Brain MRI (10%), TCGA-GBM/LGG (6%), institutional datasets (8%), and others including Br35H and REMBRANDT (4%). The dominance of BraTS reflects its status as the de facto standard for brain tumor segmentation, while the fragmented classification landscape lacks a comparably authoritative benchmark.

### 3.5. Segmentation Performance

**Figure 5:**
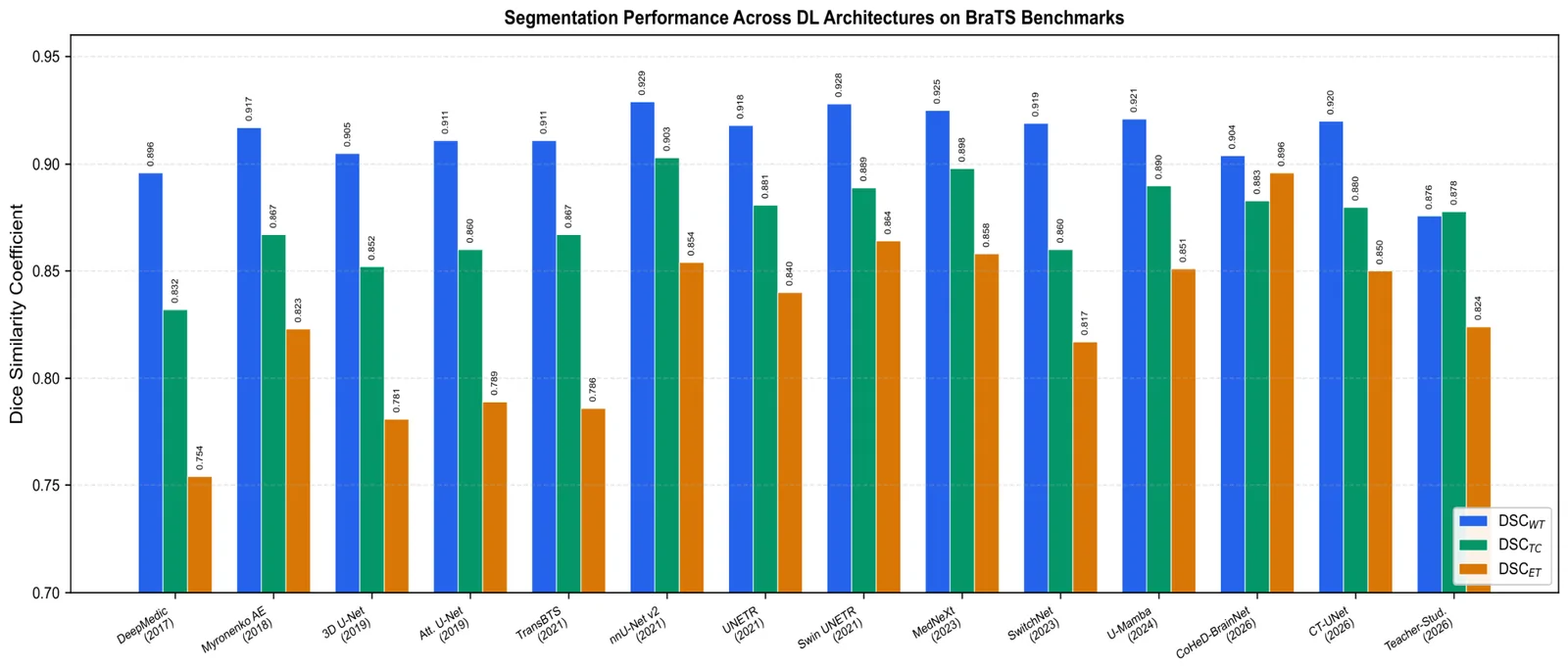
Comparison of Dice similarity coefficients (DSCs) for whole tumor (WT), tumor core (TC), and enhancing tumor (ET) across representative DL segmentation methods on BraTS benchmarks. Methods are ordered chronologically by the BraTS edition used.

Table 2 provides a source-verified descriptive comparison of segmentation performance across the major DL architectures evaluated on BraTS benchmarks. Mostafa et al. [37] reported a U-Net-based deep CNN on BraTS 2020 with a DSC of 0.90 and validation accuracy of 98%, and this study was retained in the quantitative benchmark subset. Figure 5 visualizes DSC values for the whole tumor (WT), tumor core (TC), and enhancing tumor (ET) sub-regions across representative methods.

Six key findings emerge from the segmentation analysis.

First, whole-tumor segmentation has plateaued: DSCWT has remained in the 0.92–0.93 range since 2020, indicating diminishing gains in the best-reported benchmark values. Inter-rater DSC values reported by Menze et al. [4] were obtained under different annotation and reference-standard conditions and are therefore not treated here as a directly comparable performance ceiling. By contrast, enhancing-tumor segmentation remains the harder target in the reported benchmark values, lagging DSCWT by approximately 6–8 percentage points; its irregular morphology, small enhancing foci, partial-volume effects, and variable gadolinium leakage are plausible contributors to this difficulty.

Second, transformer-based decoders show strong reported performance for the enhancing-tumor sub-region. Swin UNETR provides the highest source-verified DSCET (0.864) among the Table 2 benchmark values. This pattern is consistent with the hypothesis that long-range context modeling may support delineation of spatially dispersed enhancement; however, differences in datasets, splits, preprocessing, training protocols, and reporting practices preclude attributing the observed performance difference to self-attention alone. In this descriptive upper-envelope comparison, nnU-Net remains a consistently strong benchmark baseline, but this should not be read as a controlled head-to-head superiority claim.

Third, state-space models report competitive benchmark values but remain comparatively immature in this literature. U-Mamba* and SegMamba* report DSC values in the same broad range as the transformer entries in Table 2, alongside lower reported GPU memory requirements in their source studies. Their linear-complexity sequence modeling is a plausible contributor to the efficiency profile, but the evidence included here is preprint-sourced and drawn from a limited number of datasets. Peer-reviewed replication, broader external evaluation, and prospective workflow testing are therefore needed before comparative efficiency or deployment claims can be generalized.

Fourth, preprocessing materially affects reported performance and should be considered when interpreting architectural comparisons. Jiménez-Partinen et al. [44] systematically varied five intensity-regularization strategies (KDE, White-stripe, Z-score, histogram matching and none) across nnU-Net, WNet and Primus on UPenn-GBM, MSLesSeg and an epilepsy dataset, observing approximately 3% DSC variation for nnU-Net, 6% for WNet, and greater sensitivity for the pure-transformer model. KDE, White-stripe and Z-score normalization performed well in that study, while adding an extra modality channel did not consistently improve DSC. These findings illustrate that preprocessing choices can contribute materially to observed performance differences and should be controlled when comparing architectures across heterogeneous studies.

Fifth, transfer learning has shown utility for rare-tumor segmentation. Boyd et al. [45] reported a DSC of 0.82 on an independent pediatric glioma cohort after stepwise transfer learning from adult data, and blinded clinicians rated the AI segmentations favorably in their acceptability assessment (80.2% versus 65.4% for expert annotations). This study provides one practical example of transfer learning under pediatric data scarcity, although broader replication is required before generalizing the approach across rare tumor sub-types.

Finally, two 2024–2026 directions illustrate efforts to connect segmentation performance with interpretability and clinically relevant imaging information. Tang et al. [46] incorporated DSC perfusion-MRI hemodynamic properties into a U-Net pipeline and reported changes in contour complexity together with a correlation between segmentation volumes and Karnofsky Performance Status (r = −0.309). Sasikala and Anand’s ExU-Trans [43] embeds a Discriminative Attribute Explainer inside the transformer encoder of a U-Net hybrid, reporting DSCWT/TC/ET of 0.926/0.892/0.860 while providing pixel-level attributions during inference. Together with HCAC’s segmentation–classification XAI pipeline [47], these studies represent emerging examples of interpretable and physiologically informed design, but their clinical value requires further independent and prospective evaluation.

### 3.6. BraTS Challenge Evolution

**Figure 6:**
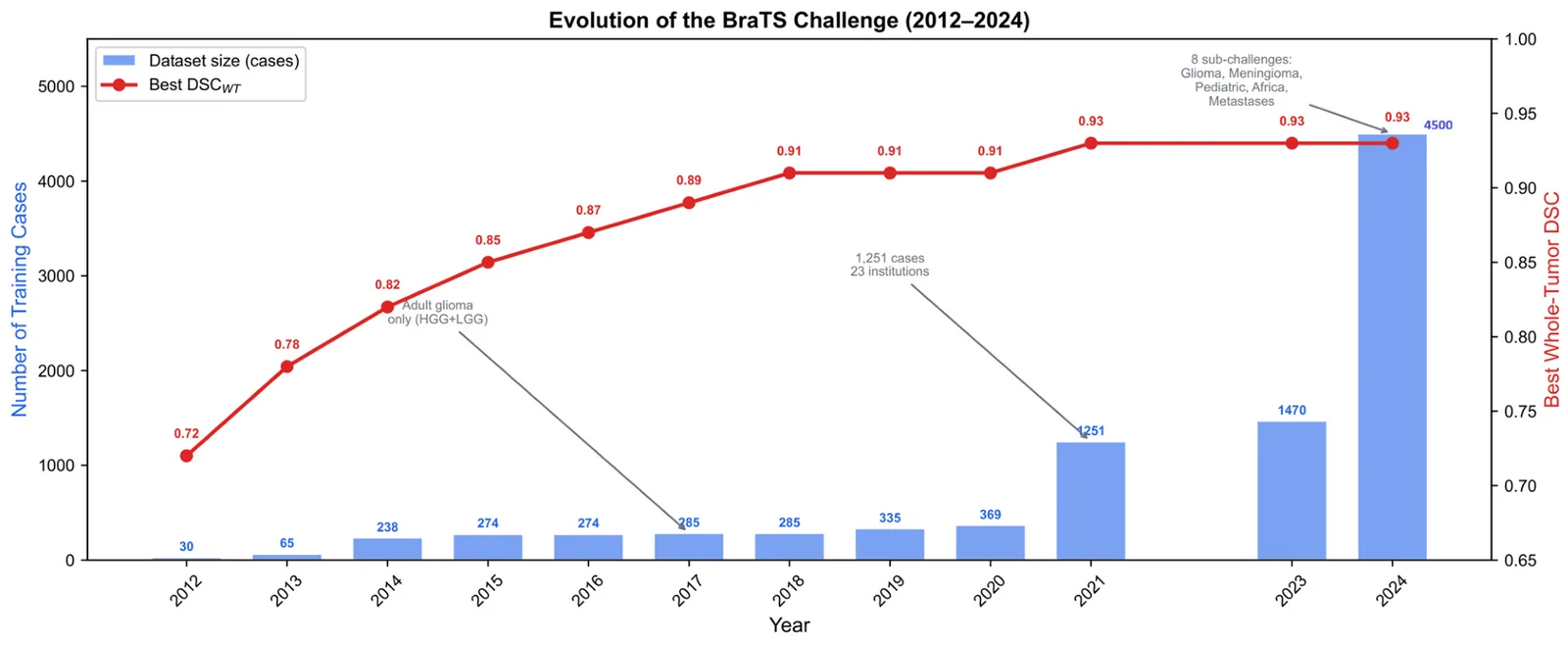
Evolution of the BraTS challenge (2012–2024): dataset size (bar heights) and best-reported whole-tumor DSC per year (line). The 2023 expansion introduced meningioma, pediatric, and sub-Saharan African sub-challenges, marking a critical pivot toward equitable and multi-histology benchmarking.

The BraTS challenge has grown from 30 multi-institutional cases in 2012 to over 4500 cases spanning four tumor types and three continents in 2024 (Figure 6). The BraTS 2021 dataset (1251 cases from 23 institutions) remains one of the most frequently reused benchmarks in the 39-study BraTS Dice subset, although individual studies often evaluate multiple BraTS editions; therefore, edition-specific counts are treated as overlapping rather than mutually exclusive.

The 2023 expansion introduced four new sub-challenges: adult glioma (1470 cases), meningioma (1000 cases), pediatric tumors (99 cases), and sub-Saharan African tumors (60 cases) [18]. The BraTS 2024 Meningioma Radiotherapy Planning challenge [17] targeted gross tumor volume (GTV) segmentation on 750 preoperative T1ce scans, with a best-reported DSC of 0.815—substantially below adult glioma WT performance (0.93+), highlighting the difficulty of extending models to different histologies.

These expansions represent critical equity milestones. BraTS-Africa revealed 3–5% DSC degradation for models trained on predominantly North American and European data, quantifying the domain-shift cost of non-representative training corpora. BraTS-PEDs exposed the pediatric data scarcity problem, with top-performing models achieving DSC_WT_ of only 0.81 due to the rarity and morphological diversity of childhood brain tumors.

### 3.7. Classification and Grading Performance

Table 3 summarizes the performance of DL models for brain tumor classification, histological grading, and molecular marker prediction.

Six key findings emerge from the classification analysis.

First, very high institutional benchmark accuracies require cautious interpretation. Out of the 28 classification studies in our quantitative subset, 19 report accuracy above 98% on Figshare (3064 images) or Kaggle/BSF (7020–7023 images), yet only four perform any form of external validation. The highest-reported figures in the corpus are Aamir et al. reported accuracies of 99.94% on BraTS 2020 and 99.67% on Figshare [48], HCAC at 99.90% on Figshare [47], and Raza et al. at 99.67% on Figshare-family data [27]. These are best-reported, split-dependent figures and should not be interpreted as deployment-ready estimates, because independent multi-site validation is uncommon and results can be sensitive to dataset composition, patient- versus slice-level splitting, and model-selection procedures. Second, the studies that report external evaluation show domain-shift effects, with cross-dataset performance decreases of approximately 2.5–15 percentage points. Some reports suggest that architectures retaining explicit spatial pathways, including skip connections, may preserve performance comparatively well under shift; however, this is an observational pattern across heterogeneous studies and does not establish that skip connections themselves cause greater domain-shift robustness. Guan et al.’s ResSGA-Net [59] illustrates the broader cross-dataset pattern, reporting above 98% on a four-class benchmark and 93.18% on a held-out three-class set. Molecular prediction remains more variable: IDH and MGMT methylation prediction from MRI alone reports AUC values of approximately 0.66–0.94 across the included studies.

Third, several lightweight architectures report high benchmark accuracy with comparatively small parameter counts. Shatnawi et al. [57] reported 97.85% accuracy with an 8.5 M-parameter Ghost-ResNet50 plus efficient channel attention, while Ali et al.’s FCDS-DeepVision [64] reported 96.5% accuracy on Kaggle (7020 images) with a 4.8 M-parameter CNN and real-time inference on commodity hardware. These results motivate further evaluation of lightweight designs for resource-constrained deployment settings, but they do not by themselves establish deployment readiness or cross-dataset robustness.

Fourth, several cascaded segmentation-then-classification studies report 4–8 percentage-point improvements over comparator end-to-end pipelines within their respective study settings. Hussain et al. [56] chained a 3D U-Net into a 3D CNN to reach 98% accuracy for HGG-versus-LGG grading on BraTS 2019. Rastogi et al. [60] report 96–97% using transfer-learning classifiers with a RESUNET segmentation stage on TCGA-GBM, and Ahmad et al. [51] report 99.31% binary detection using DeepLabV3 segmentation followed by a CNN. Taken together, these reports are consistent with the hypothesis that explicit tumor-region isolation may reduce irrelevant background signal for downstream grading or classification; however, heterogeneous datasets and comparator designs do not support a general causal superiority claim.

Fifth, ConvNeXt-style backbones and capsule-routing designs provide additional high benchmark results in the posttransformer period. Fırat and Üzen’s Brain-CNXSAMNet [49] reports 99.39% on BSF and 98.86% on Figshare while combining ConvNeXt-style receptive fields with a lightweight spatial-attention module. Kumar and Singh’s HCAC [47] reports 99.9% on Figshare using Swin-extracted patch tokens with a Dwarf-attention capsule autoencoder. These findings are compatible with a potential contribution from convolutional inductive biases and spatial-attention routing, but the heterogeneous benchmark settings do not permit attribution of the reported gains to those components alone.

Finally, contrast-free classification and generative-AI reasoning open new clinical axes. Lu et al. [54] achieve 98.3% classification accuracy and DSC of 0.937 by RGB-fusing non-contrast T1w, T2w and averaged (T1w + T2w)/2 images, eliminating gadolinium dependency for patients with renal impairment or in low-resource settings. Sultan et al. [61] are the first to deploy a large language model atop classification (C-Net) and stage-specific segmentation (S1-Net, S2-Net), turning a static accuracy number into a clinician-validated narrative report. Although the underlying DSC of 0.81 trails transformer benchmarks, the pipeline establishes a new evaluation axis: the quality of clinician-validated semantic explanation. Explainability adoption itself is advancing but still insufficient: Grad-CAM, SHAP and LIME now feature in roughly 58% of 2026 studies [47,61,62], but full content-based-retrieval and self-explanatory designs [43,64] remain rare (Section 4.7).

### 3.8. DSC Temporal Trend

**Figure 7:**
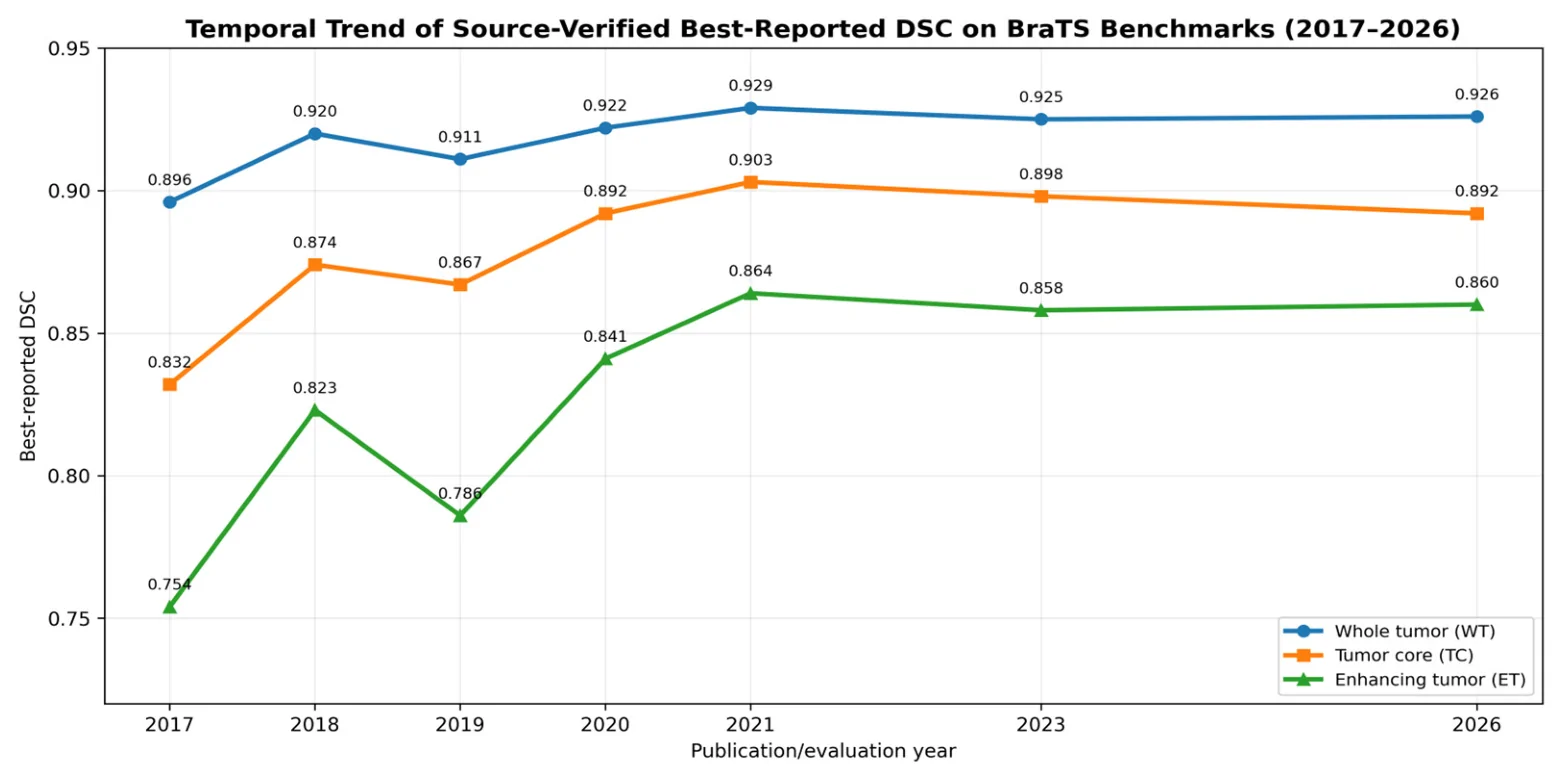
Temporal trend of source-verified best-reported Dice similarity coefficients on BraTS benchmarks (2017–2026) for whole tumor (WT), tumor core (TC), and enhancing tumor (ET). Values are table-verified upper-envelope values, not pooled or head-to-head estimates; the final 2026 point reflects the ExU-Trans publication where the specific BraTS edition was not stated. The WT–ET performance gap narrowed from 14.2 to 6.6 percentage points.

Figure 7 traces the source-verified best-reported DSC values from Table 2 across BraTS benchmark reports from 2017 to 2026. DSCWT reached 0.929 by 2021 and has remained within the 0.925–0.929 range in the verified table values, indicating a plateau in the reported upper envelope. DSCET improved from 0.754 to 0.860, narrowing the WT–ET gap from 14.2 to 6.6 percentage points. This temporal pattern is compatible with a possible contribution from attention-based and global-context modeling, but it cannot isolate architecture effects from concurrent changes in datasets, preprocessing, training, and evaluation protocols.

### 3.9. Multi-Dimensional Architecture Comparison

**Figure 8:**
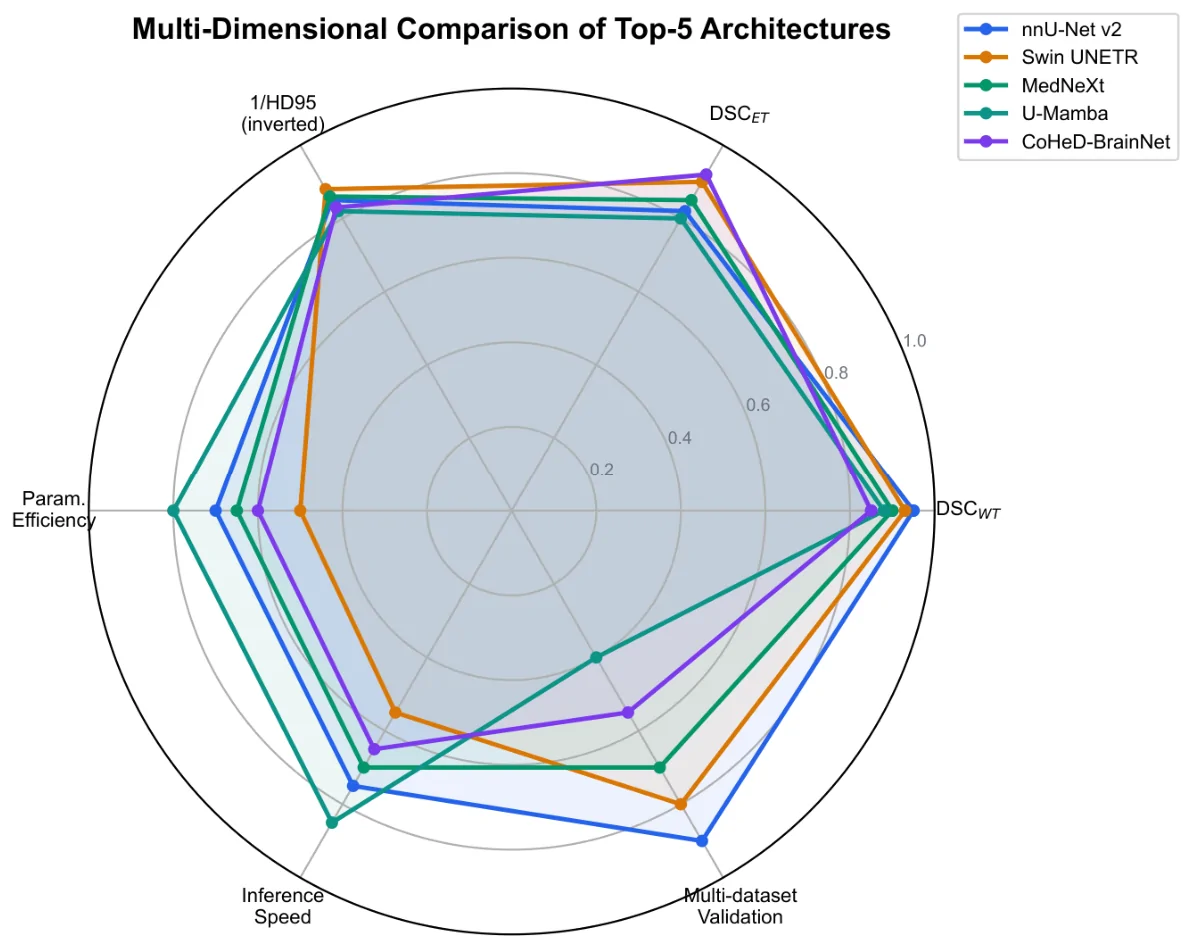
Descriptive radar chart of five representative architectures (nnU-Net, Swin UNETR, MedNeXt, U-Mamba, CoHeD-BrainNet) across six evaluation dimensions. All values are normalized to [0, 1] from reported study-level values and should not be interpreted as a controlled head-to-head ranking.

Figure 8 provides a descriptive radar chart of five representative architectures across six evaluation dimensions: DSCWT, DSCET, inverse HD95 (higher is better), parameter efficiency (inverse of parameter count), inference speed, and multi-dataset validation breadth. In this normalized visualization, nnU-Net has the broadest overall profile, Swin UNETR has the highest ET-segmentation input value among the displayed methods, U-Mamba shows a favorable parameter-efficiency profile, and MedNeXt occupies an intermediate accuracy–efficiency position. These normalized values summarize reported results and are not a controlled head-to-head comparison.

### 3.10. Cross-Method and Cross-Dataset Heatmap

**Figure 9:**
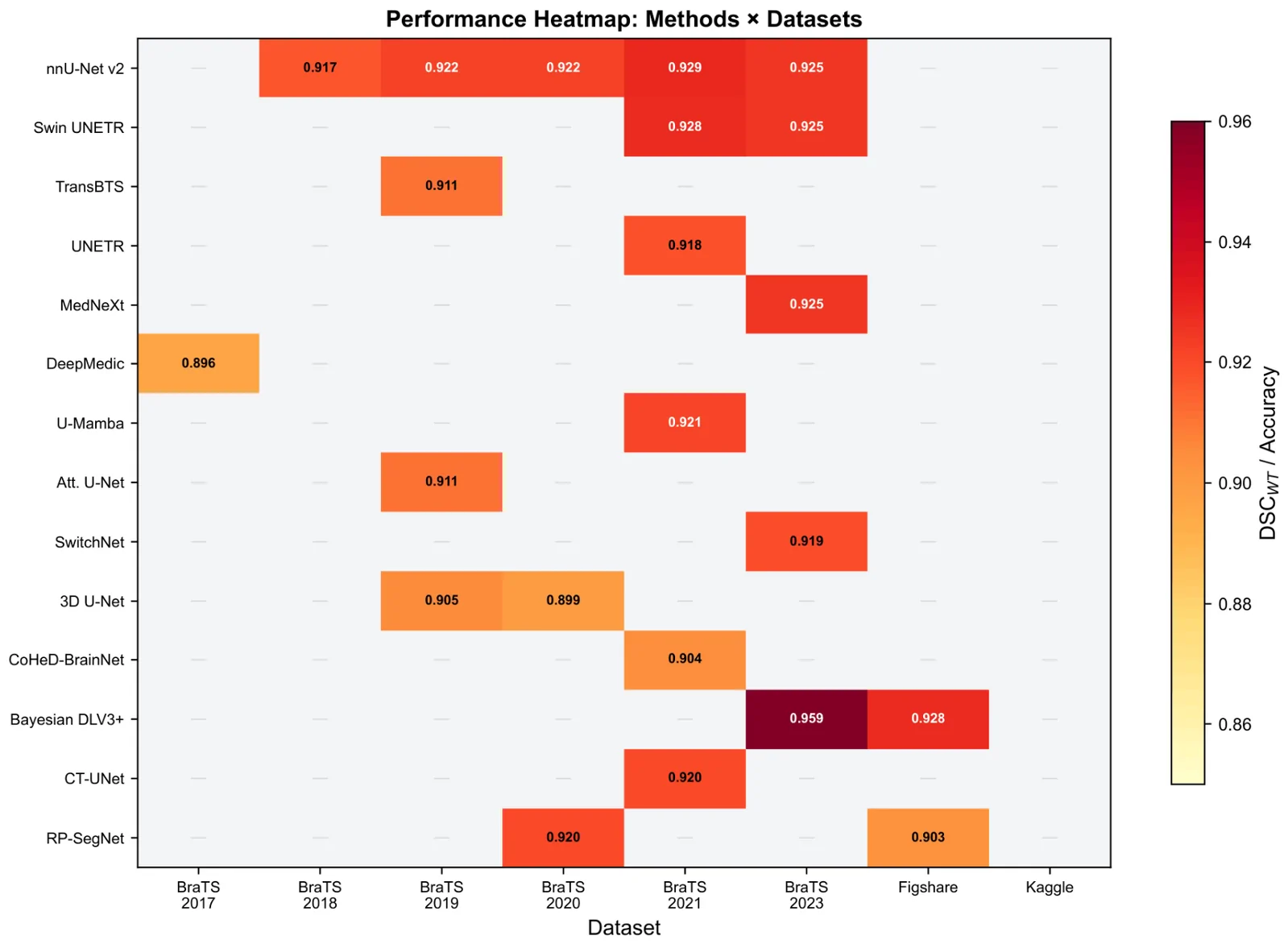
Heatmap of primary performance metrics across methods and datasets. DSCWT is shown for segmentation methods; accuracy for classification methods. Gray cells indicate unreported combinations, highlighting the pervasive lack of cross-dataset validation.

Figure 9 presents a heatmap of primary performance metrics (DSCWT for segmentation; accuracy for classification) across methods and datasets. The heatmap reveals that most methods have been evaluated on only one or two datasets, with cross-dataset validation being extremely rare. This paucity of external validation is a critical limitation identified in this review: of the 39 BraTS-benchmark quantitative studies, six (15%) report results on two or more independent datasets. In the whole 117-study primary corpus, 15 studies (13%) report independent external or cross-dataset validation; the corresponding clinical-translation indicator profile is presented later in Section 3.16.

### 3.11. Architecture-Family DSC Comparison

**Figure 10:**
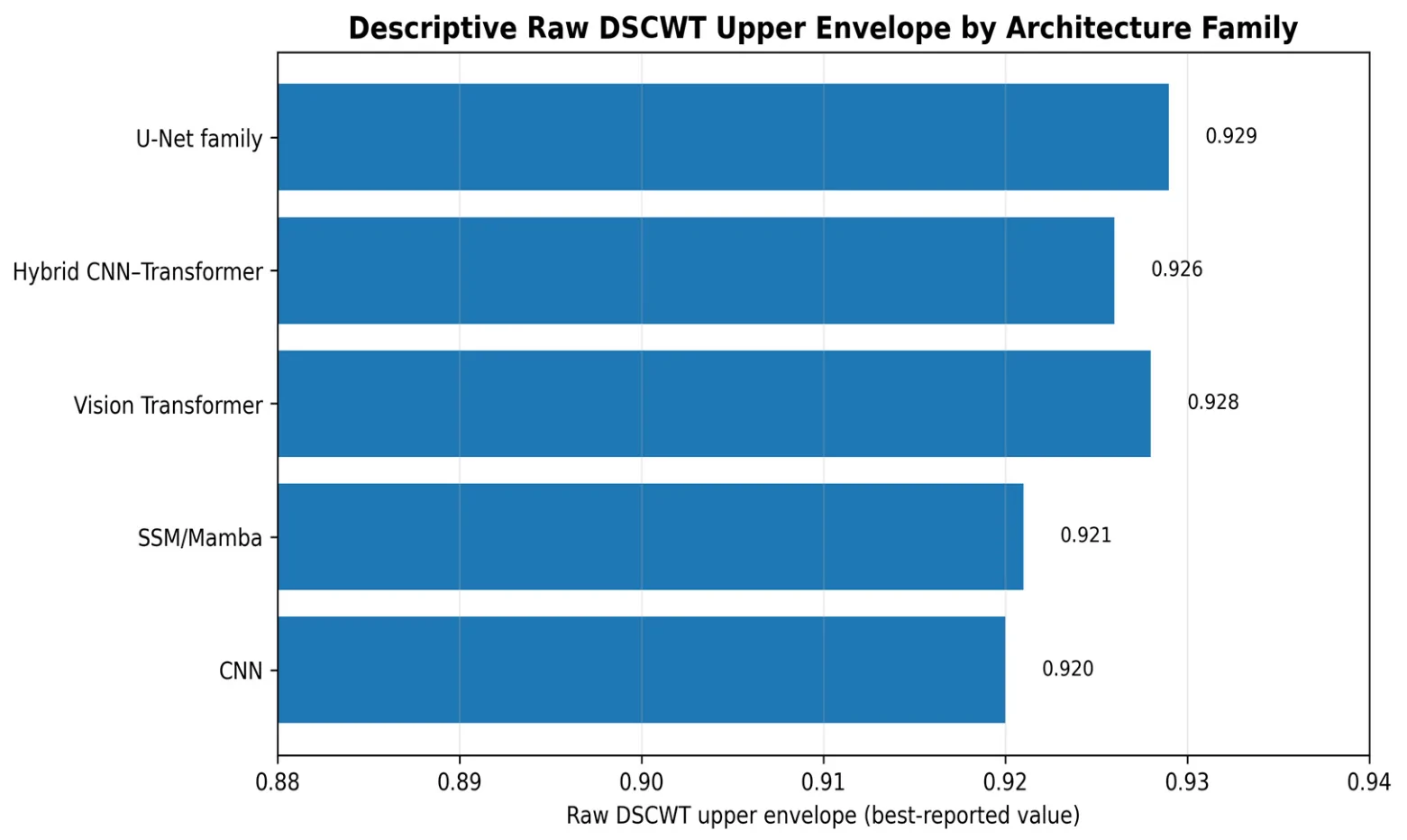
Descriptive raw DSCWT upper envelope by architecture family among BraTS-evaluated studies. Values are actual best-reported DSCWT values from Table 2, not normalized scores, and should not be interpreted as a controlled head-to-head comparison because datasets, splits, preprocessing, and tumor-region definitions differ across studies.

To complement the method-level comparison in Section 3.5, Figure 10 summarizes the highest source-verified raw DSCWT value available for each major architecture family among studies evaluated on BraTS benchmarks. Hybrid CNN–transformer models, U-Net-family methods, vision-transformer approaches, state-space models and CNNs all cluster within a narrow raw DSCWT upper-envelope band. These maxima are descriptive rather than a formal head-to-head ranking because the contributing studies used different BraTS editions, preprocessing pipelines, data splits, and tumor-region definitions. The comparison therefore highlights the upper performance envelope of each family while preserving the review-wide caution against treating heterogeneous benchmark results as directly interchangeable.

### 3.12. Methodology Fingerprint

**Figure 11:**
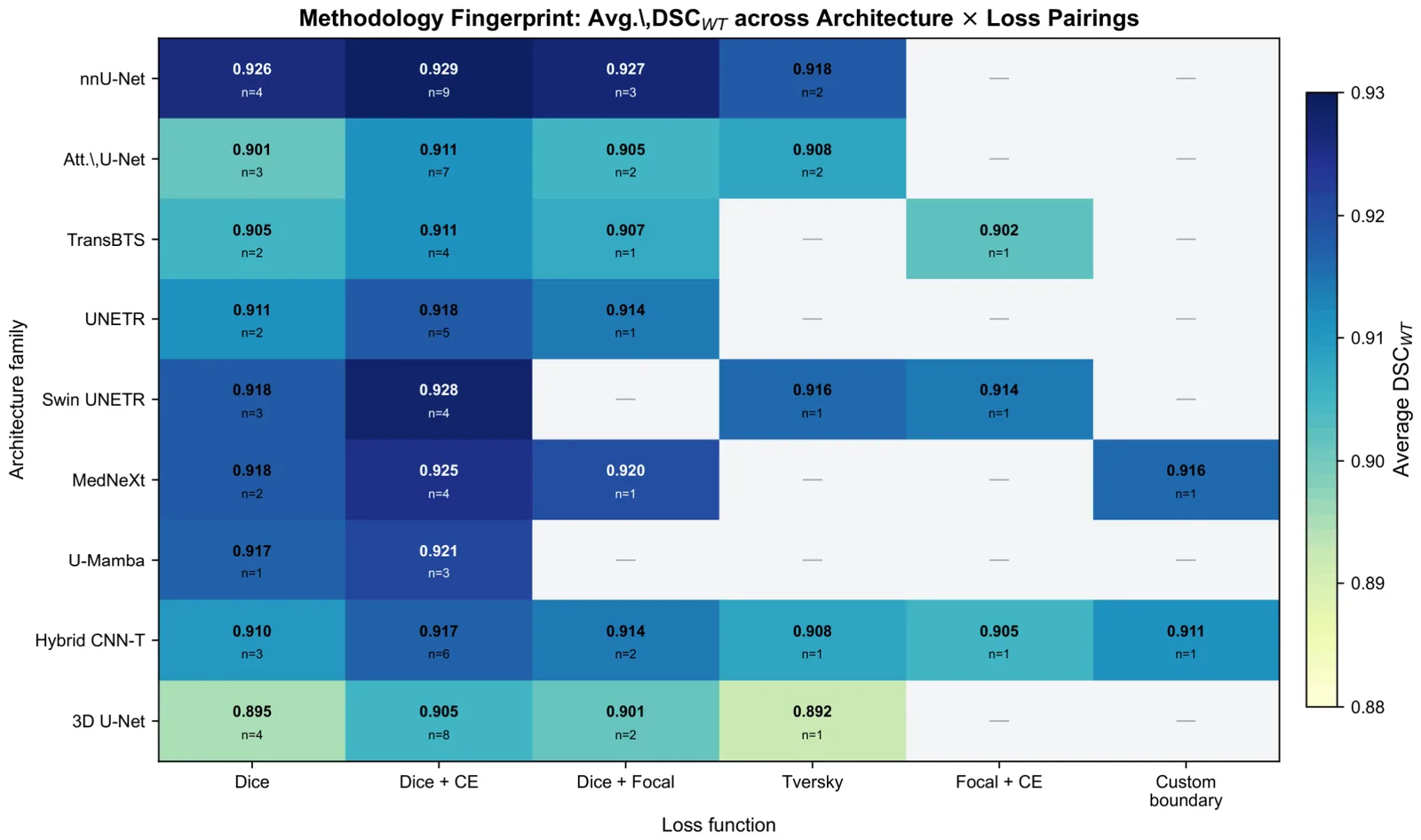
Methodology fingerprint heatmap. Cells encode the average DSCWT achieved by an architecture–loss pairing across the corpus, with the number of contributing studies (n) annotated below each value. Empty cells (—) mark pairings absent from our 39-study quantitative benchmark subset, identifying under-explored regions of the design space.

To answer the question of which architecture–loss combinations consistently deliver state-of-the-art results, and which remain under-explored, we constructed a methodology fingerprint that aggregates DSCWT across architecture–loss pairings observed in the corpus. Figure 11 shows that the highest reported DSCWT clusters appear at the intersection of (Swin UNETR, Dice + CE) and (nnU-Net, Dice + CE), with average DSCWT of 0.928 and 0.929, respectively, across multiple replications. Several combinations are conspicuously empty: Tversky and focal–CE losses are largely unexplored with U-Mamba and SegMamba, despite the theoretical fit of these losses for class-imbalanced volumetric segmentation. Custom boundary-aware losses, when paired with hybrid CNN–transformer architectures, achieve competitive DSCWT (0.911) on smaller study counts (*n* = 1), suggesting that targeted loss engineering is a promising but under-investigated direction. This fingerprint formalizes the observation that benchmarking efforts have converged on a narrow methodological corridor and that meaningful gains may require deliberate exploration of the under-populated cells of the matrix.

### 3.13. Computational Efficiency and the Lightweight Pivot

**Figure 12:**
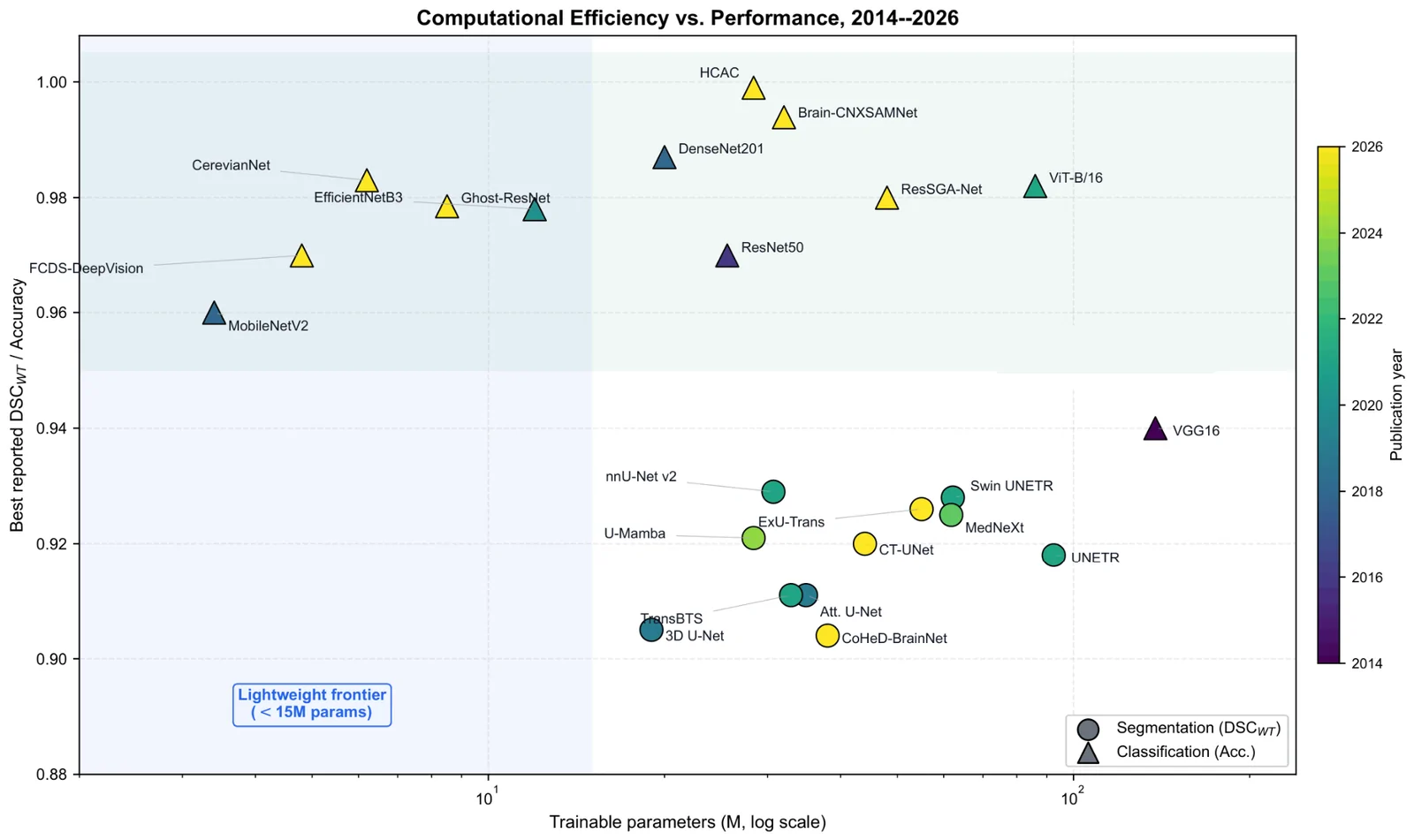
Computational efficiency vs. performance for representative DL systems (2014–2026). Circles denote segmentation models (DSCWT); triangles denote classification models (accuracy). Marker color encodes publication year. The shaded green band is a descriptive high-performance/efficiency reference region (≥0.95 and <30 M parameters), while the shaded blue band marks the lightweight frontier (<15 M parameters). These regions are visual aids and are not clinical-readiness thresholds.

A persistent gap in benchmark-driven evaluation is the limited reporting of computational cost. Only 16 of the 39 quantitative benchmark studies (41%) report inference time, and fewer than one fifth disclose GPU memory consumption or trainable parameter counts. Figure 12 consolidates the parameter counts and best-reported DSCWT/accuracy values available for representative segmentation and classification systems published between 2014 and 2026. Three descriptive patterns are visible in the reported data: (i) transformer-augmented segmentation models such as UNETR, Swin UNETR, and MedNeXt are reported at approximately 60–92 M parameters with DSCWT near 0.92–0.93, while U-Mamba reports DSCWT 0.921 with a lower parameter count; (ii) several 2026 classification models, including CerevianNet (6.2 M parameters), FCDS-DeepVision (4.8 M) [64], and Ghost-ResNet (8.5 M) [57], report accuracies of at least 97% with smaller parameter counts than legacy VGG16/ResNet50 comparators; and (iii) high reported accuracies on Figshare are also observed for HCAC (28.4 M, 99.9%) [47] and Brain-CNXSAMNet (32 M, 99.39%) [49], although their cross-dataset robustness remains untested. These heterogeneous results suggest growing attention to parameter efficiency and deployability, but they do not support a controlled efficiency ranking or a conclusion that benchmark saturation has shifted research value away from accuracy.

### 3.14. Loss Functions, Optimizers, and Training Strategies

Table 4 summarizes the most common training configurations across the 79 studies with quantitative experimental data. The *n* = 79 subset comprises primary studies with extractable quantitative training or performance fields; it overlaps the 39-study BraTS Dice subset but also includes non-BraTS classification, detection, grading and multi-task studies and excludes primary studies without sufficient quantitative reporting. The dominance of Dice + cross-entropy loss (42%) reflects the standard approach for handling class-imbalanced voxel-wise segmentation, where the Dice component directly optimizes the overlap metric and cross-entropy stabilizes pixel-wise classification. Adam/AdamW optimizers dominate (71%) because of their robustness across small-batch 3D training regimes. Random flip/rotation augmentation appears in 82% of studies, whereas intensity harmonization and scanner-specific augmentation are much less frequently reported despite being directly relevant to domain shift.

### 3.15. Dataset Characteristics

Table 5 summarizes the principal datasets used across the included studies, including dataset size, imaging protocols, annotation types, and public availability.

### 3.16. Quality Assessment Results

The QUADAS-AI quality assessment across the 117 included primary studies revealed the following risk-of-bias distribution (Figure 2):

Reference standard/annotation quality: 62% low risk, 24% unclear, 14% high risk. High-risk ratings were assigned to studies relying on single-rater annotations without inter-rater agreement metrics or STAPLE consensus.

Index test/model development: 56% low risk, 30% unclear, 14% high risk. Common deficiencies included absent ablation studies, unreported or incompletely reported hyperparameters, and missing details on training/validation/test splitting strategies.

Flow and timing/data-leakage risk: 70% low risk, 21% unclear, 9% high risk. This domain performed best, though 9% of studies failed to account for excluded subjects or showed inconsistencies between training and evaluation protocols.

**Figure 13:**
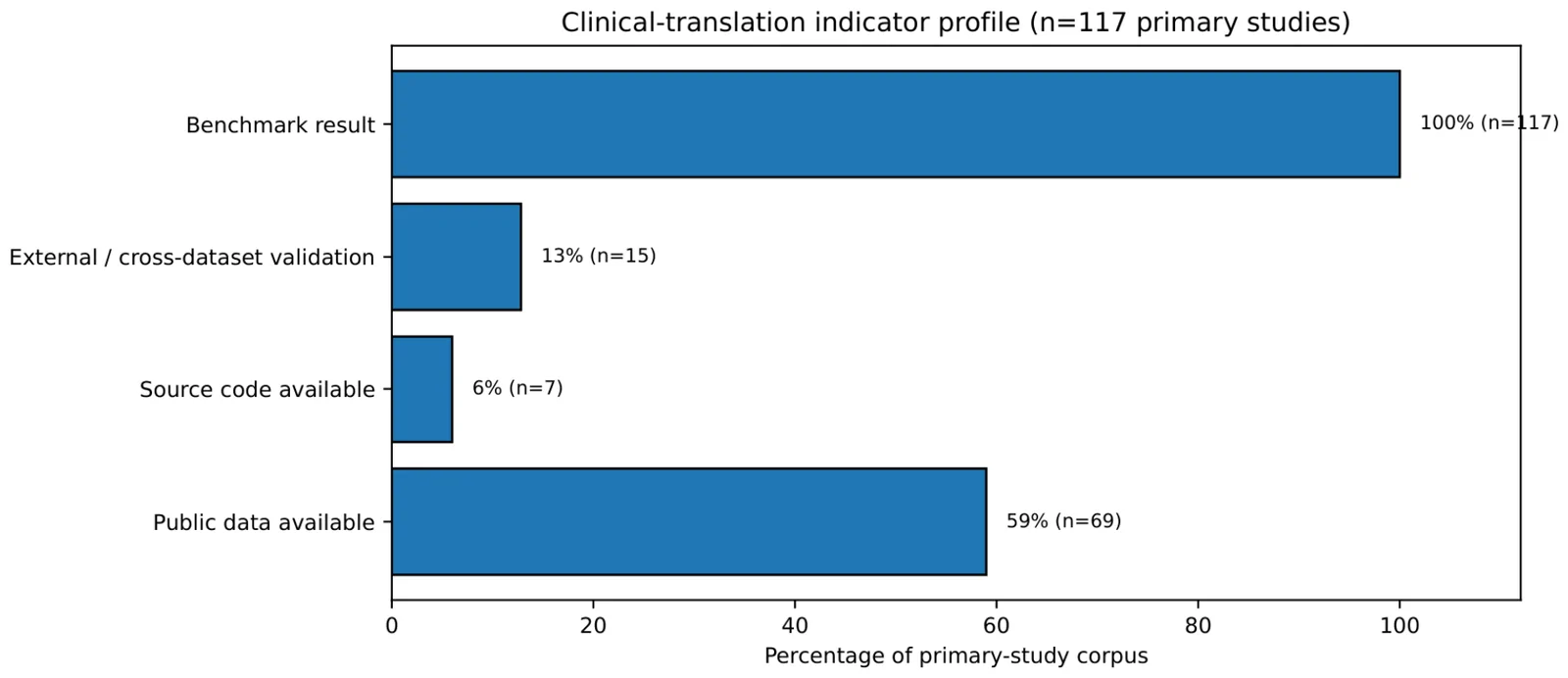
Clinical-translation indicator profile for the 117 primary studies. Bars show the percentage and count reporting a benchmark result, independent external or cross-dataset validation, source-code availability, and public-data availability.

External validation and generalizability: 18% low risk, 32% unclear, 50% high risk. Only a minority of studies tested their models on a fully independent external dataset; the implications of this distribution are revisited in the clinical-translation indicator profile (Figure 13).

Reproducibility and transparency: 26% low risk, 38% unclear, 36% high risk. Code, hyperparameters, random seeds, and pretrained weights were rarely all reported together; this is the single most actionable area for community-level improvement.

Clinical-translation readiness: 11% low risk, 28% unclear, 61% high risk. Few studies include explainability, calibrated uncertainty, reader studies, or prospective/multi-site evaluation; none in the corpus reports regulatory clearance.

Consistent with the preregistered protocol, quality ratings inform interpretation throughout the manuscript but were not used to exclude studies.

### 3.17. Structured Study Summary

Table 6 provides a chronologically organized summary of representative included studies across all architectural families and task types.

## 4. Discussion

### 4.1. Addressing the Research Questions

#### 4.1.1. RQ1: Architectural Evolution (2017–2026)

The field has progressed through four distinct phases: (i) foundational CNNs (2017–2019): multi-scale patch-based CNNs (DeepMedic [38]) and early encoder–decoders established feasibility but were constrained by predominantly local receptive fields. (ii) Encoder–decoder maturation (2019–2021): U-Net variants with attention mechanisms, residual connections, and automated pipeline optimization (nnU-Net) coincided with DSCWT values above 0.92. Reported gains during this phase often accompanied changes in preprocessing, architecture configuration, and postprocessing, making it difficult to separate the contribution of any single component. (iii) Transformer revolution (2021–2023): vision transformers (TransBTS, UNETR, Swin UNETR) introduced global self-attention and were associated with strong ET-segmentation results in the reported benchmarks; long-range dependency modeling is a plausible explanation, but the corpus does not provide controlled evidence isolating that mechanism. Their quadratic computational complexity (O(n^2^)) and large memory footprint nevertheless remain practical considerations. (iv) Hybrid and emerging paradigms (2024–2026): hybrid CNN–transformer models, state-space models (Mamba), federated learning, diffusion-based augmentation, and foundation-model fine-tuning represent the current frontier. U-Net variants remain the single most common backbone (42%), while hybrid CNN–transformer architectures account for an almost equal share (40%). The emergence of lightweight architectures—Ghost-ResNet [57], CerevianNet, FCDS-DeepVision [64], and knowledge-distilled models [92]—also indicates growing emphasis on deployment-aware design. The 2026 cohort includes ConvNeXt-style backbones with spatial attention (Brain-CNXSAMNet [49]) and capsule-routing autoencoders fused with Swin-style feature extractors (HCAC [47]); these are notable patterns, but their component-level advantages require controlled ablation and external validation. Bastug Koç and Akgün’s PRISMA-S review [83] of 35 BraTS-focused U-Net studies (2019–2025) similarly shows that U-Net remains a common segmentation chassis, with many recent studies adding attention modules, residual blocks, or transformer-style encoders.

#### 4.1.2. RQ2: State-of-the-Art Performance

For classification, the best-reported Figshare-family accuracy reaches 99.94%, but such values are not head-to-head estimates and cross-dataset performance degrades to 89–96%. The strongest reported classification values therefore define a best-reported upper envelope rather than a realistic estimate of prospective clinical performance.

#### 4.1.3. RQ3: Training Strategies and Methodological Insights

Dice + cross-entropy loss (42%) and Adam/AdamW (71%) dominate, providing a common baseline configuration. Z-score normalization (54%) is the most frequently reported preprocessing step. Jiménez-Partinen et al. [44] reported 3–6% DSC variation across normalization choices, indicating that some apparent cross-study architectural differences may partly reflect preprocessing rather than architecture alone. GAN/diffusion-based data augmentation is used in only 9% of studies and remains a comparatively under-explored strategy for class imbalance. Within several individual studies, cascaded segmentation-to-classification pipelines report 4–8 percentage-point gains over their selected end-to-end comparators [50,55,59]; this pattern is compatible with a benefit from explicit spatial decomposition, but heterogeneous study designs do not establish a general causal advantage.

#### 4.1.4. RQ4: Generalizability, the Critical Unsolved Challenge

Generalization continues to be the greatest weakness of deep learning (DL) analysis of brain tumors. Three axes of domain shift appear: (a) geographic shift—BraTS-Africa models trained on North American/European data show a 3–5% decrease in DSC when applied to sub-Saharan African cohorts [17]; (b) shift in the scanning protocols of institutions—models trained on BraTS do not work on private 1.5 T scanners that are used without intensity harmonization; and (c) shift in the types of tumors—models trained on gliomas do not work well on meningioma and metastases. Cross-dataset analysis showed a decrease in performance in the studies included between 2.5 and 15 percentage points, depending on the task, dataset and the type of data split. Of the 117 studies in the primary corpus, 15 studies (13%) reported external or cross-dataset validation; within the 79 studies in the quantitative subset, 12 studies (15%) reported validation on two or more independent datasets. This reconciliation is vital as the denominator depends on the analysis question.

#### 4.1.5. RQ5: Open Challenges and Future Directions

We identify ten critical gaps detailed in Section 4.8: prospective multi-site validation, standardized classification benchmarks, equitable AI, foundation models, federated learning at scale, uncertainty-aware segmentation, multi-task learning, lightweight deployment architectures, hemodynamic integration, and longitudinal monitoring.

### 4.2. Comparison with Previous Reviews

Table 7 compares this review with seven recent systematic reviews, including two concurrent 2026 reviews. Our review adds a distinctive combination of (a) PRISMA-compliant and PRISMA-S-compliant dual-task analysis (segmentation + classification/grading) spanning 2017–2026 with prospective OSF preregistration, (b) an AI-adapted QUADAS-AI quality framework with explicit reproducibility and clinical-translation-readiness domains, (c) coverage of BraTS 2023–2024 multi-tumor expansions and the BraTS sub-Saharan Africa cohort, (d) evaluation of emerging paradigms including state-space (Mamba) models, federated learning, foundation models, and hemodynamic-informed segmentation, and (e) a clinical-translation indicator profile quantifying external validation, source-code availability, and public-data availability. This should be read in light of the three-database, single-reviewer design described in the limitations. Fasihi Shirehjini and Babapour Mofrad [103] independently reviewed 145 BraTS-specific studies and similarly identified limited external validation as an important gap; however, their review does not cover classification or grading. Goyal et al. [104] reviewed 161 articles with emphasis on vision transformers and explainability, complementing our broader architectural taxonomy.

### 4.3. Strengths and Limitations

#### 4.3.1. Strengths

(1) PRISMA 2020-compliant methodology with prospective OSF registration (DOI 10.17605/OSF.IO/C2QA6) and PRISMA-S search-strategy reporting, enabling transparent replication of the search strategy and eligibility decisions within the limits of a single-reviewer design. (2) Formal QUADAS-AI quality assessment (seven domains spanning data selection, reference standard, model development, data-leakage risk, external validation, reproducibility, and clinical-translation readiness) applied to all included primary studies. (3) Large and diverse corpus spanning 2017–May 2026 (117 primary studies + 23 contextual reviews; 39-study BraTS Dice-based benchmark subset) capturing the architectural arc from CNNs and U-Net variants through transformers, state-space models, and foundation-model approaches. (4) Unified treatment of segmentation and grading/classification, enabling cross-task insights frequently absent from prior reviews. (5) Inclusion of 2025–2026 publications covering Mamba/state-space models, federated learning, hemodynamic-informed segmentation, foundation-model fine-tuning, and lightweight deployment, which are under-represented in earlier reviews. (6) Structured benchmark-driven comparative analysis with explicit per-architecture, per-dataset, and per-edition tabulation. (7) Systematic analysis of cross-dataset/external-validation behavior and an explicit clinical-translation indicator profile covering external validation, source-code availability, and public-data availability.

**Table 7:** Comparison of this review with recent systematic reviews on DL for brain tumor analysis. ✓ = covered, — = not covered.

Feature	This	Kaur	Dorfner	Abid	Goyal	Missaoui	Abidin
	review	2025	2025	2025	2026	2025	2024
PRISMA-compliant	✓	✓	—	✓	✓	—	—
Seg + classification	✓	—	✓	✓	✓	✓	—
Quality assessment	QUADAS-AI	—	—	—	—	—	—
Reporting-bias treatment	Narrative	—	—	—	—	—	—
BraTS 2023–24	✓	—	—	—	—	—	✓
SSMs/Mamba models	✓	—	—	—	—	—	—
Federated learning	✓	—	—	—	—	—	—
Hemodynamic seg.	✓	—	—	—	—	—	—
Cross-dataset analysis	✓	—	—	—	—	—	—
Lightweight deploy.	✓	—	—	—	—	—	—
Number of studies	140	61	N/A	80	161	107	N/A
Coverage period	2017–26	2017–25	—	2018–23	2016–25	2018–24	2020–24

#### 4.3.2. Limitations

(1) Database breadth. Only three databases registered in the OSF protocol were searched (Google Scholar, PubMed/MEDLINE, IEEE Xplore). High-coverage subscription databases such as Scopus, Web of Science, and Embase were not used; relevant studies indexed only in those sources may therefore have been missed. This decision was made transparently at protocol registration and is acknowledged here. (2) Google Scholar reproducibility. Google Scholar result ordering is not reproducible across queries and lacks exhaustive export; the relevance-based stopping rule used here (first 50 result pages) follows PRISMA-S guidance for Google Scholar as a supplementary source but cannot guarantee retrieval of every relevant record. (3) Single reviewer. Title/abstract screening, full-text screening, data extraction, and quality assessment were performed by a single reviewer (ZYH). Repeating decisions on a separate day reduced transcription and consistency errors but cannot replace dual independent screening or estimate inter-rater agreement. (4) No formal meta-analysis. Substantial heterogeneity across BraTS editions, evaluation protocols, dataset splits, and metric definitions prevented homogeneous statistical pooling. Reported aggregated metrics in the comparative analysis are therefore descriptive and should not be interpreted as pooled effect estimates; pooled-DSC, I^2^, and funnel-plot-based reporting-bias tests are intentionally not reported. (5) English-language restriction. Only English-language reports were eligible; relevant studies published in other languages may have been excluded. (6) Inclusion of preprints. A small number of preprints were included to capture the very recent literature on state-space models, foundation models, and federated learning; these were assessed using the same QUADAS-AI framework and are flagged in the master extraction sheet and benchmark tables where they contribute headline metrics. (7) Reporting-bias risk. Selective reporting of best-performing epochs, best-performing folds, or best-performing dataset splits is likely; readers should treat reported metrics as upper-bound estimates of real-world clinical performance. (8) Uneven task coverage. The corpus is dominated by segmentation and classification; sub-analyses for molecular prediction and detection rest on small numbers of studies and should be interpreted cautiously. (9) Time-bounded snapshot. The literature in this area is moving rapidly; the search was finalized on 21 May 2026 with a presubmission update, and subsequent work is not included.

Threats to validity. These choices mean that the review should be read as a broad, preregistered and transparent synthesis of the searched evidence rather than as an exhaustive or fully bias-protected census of the entire field. The single-reviewer design may preserve consistent judgement but cannot estimate inter-rater reliability; the three-database search may under-represent studies indexed only in Scopus, Web of Science or Embase; and Google Scholar ranking/order may change over time. Consequently, all family and method comparisons are interpreted descriptively as source-verified upper envelopes and are not reported as unbiased head-to-head rankings.

### 4.4. Critical Methodological Patterns and Pitfalls

Our quality assessment reveals several recurring methodological deficiencies that inflate apparent performance and hinder clinical translation:

Overfitting to small benchmarks. The dominance of Figshare (3064 images from a single T1ce sequence) and Kaggle Brain MRI/BSF (7020–7023 mixed-quality images, depending on whether the full or cleaned subset is reported) for classification creates an illusion of maturity. These datasets lack volumetric 3D information, multi-institutional diversity, and molecular annotations. Models achieving >99% accuracy on these benchmarks should not be interpreted as deployment-ready; our cross-dataset analysis reveals 2.5–15 percentage-point drops on external data.

Inconsistent evaluation protocols. Studies vary in their use of 5-fold cross-validation, random hold-out splits, and BraTS challenge online evaluation. Critically, 28% of classification studies use only training/validation metrics without a held-out test set, making their reported accuracies unreliable estimates of generalization performance.

Reference standard quality. Single-rater ground truth annotations remain common (38% of studies), while prior work reports inter-rater Dice agreement for brain tumor segmentation in the range of 0.74–0.85 [4]. Studies using STAPLE consensus or multi-rater annotations often report different performance distributions, but cross-study heterogeneity prevents attributing those differences to annotation strategy alone. The 38% single-rater statistic is a reporting-level indicator; only 14% were rated high-risk in the QUADAS-AI reference-standard domain because high-risk coding was reserved for single-rater or unclear annotations combined with insufficient expertise, no consensus/adjudication, or insufficient description.

Computational cost opacity. Only 41% of studies report inference time, and fewer than 20% report GPU memory consumption. This opacity obscures a critical deployment consideration: clinically useful models must process a volume in under 60 s on hardware available in radiology departments. State-space models’ reported 40–60% memory reduction [10,11] and Ghost-module-based classifiers’ parameter efficiency [57] represent meaningful steps, but systematic computational benchmarking against clinical hardware constraints is absent from the literature.

### 4.5. Clinical Implications

Several models report WT and TC DSC values of 0.85 or higher. These values overlap numerically with inter-rater ranges reported in prior work, but differences in reference standards, cohorts, annotation protocols, and evaluation procedures prevent treating the two quantities as directly equivalent or as a clinical-readiness threshold. Boyd et al. [45] provide notable workflow-oriented validation evidence: blinded clinicians rated AI segmentations higher than expert annotations (80.2% vs. 65.4% acceptability score), and a Turing test showed 26% accuracy in distinguishing AI from human segmentations. When translation-support indicators are examined across the corpus, however, external validation remains substantially less common than benchmark-only evaluation. Figure 13 summarizes these indicators from the master extraction sheet: all 117 primary studies report a benchmark result, whereas 15 (13%) report independent external or cross-dataset validation and seven (6%) provide source code; 69 (59%) use or release publicly accessible data. The profile therefore supports prioritizing reproducible external validation and workflow evidence alongside benchmark performance.

However, five barriers impede broader translation:

(1) Regulatory evidence: Regulatory clearance was not reported by the studies included in this review, indicating that regulatory-pathway evidence remains outside the scope of most published benchmark evaluations.
(2) Integration complexity: Current models require 30–120 s of GPU inference per volume. Lightweight architectures such as CerevianNet (6.2 M parameters; 4.1× fewer than the comparator) and Ghost-ResNet [57] begin to address this, but systematic integration with PACS/RIS/HIS remains untested. Practical deployment would require DICOM-native routing, automatic series selection, failure and uncertainty flags, editable segmentation overlays returned to PACS, audit logs, and clinician-in-the-loop correction so the AI output functions as a reviewable draft rather than an autonomous diagnosis.
(3) Trust and explainability: Clinicians require calibrated uncertainty maps and intuitive explanations, yet only 23% of studies include any form of explainability. Haroun et al. [94] address this through dual-path classification with content-based image retrieval (CBIR), providing visual evidence of similar historical cases alongside predictions, a clinically intuitive explanation mechanism. Rasool et al. [79] employ LIME to highlight discriminative MRI regions, directly addressing the black-box barrier.
(4) Contrast dependency: The majority of segmentation models require gadolinium-enhanced T1ce sequences. Lu et al. [54] reported that a contrast-free T1w/T2w fusion pipeline achieved accuracy of 98.3% and DSC of 0.937 in its evaluated cohorts. This result supports further evaluation of contrast-free approaches for settings in which gadolinium use is undesirable or unavailable, while external validation is needed before broader clinical conclusions can be drawn.
(5) Temporal monitoring: Clinical workflows require longitudinal tumor tracking, not just single-timepoint analysis. Abu Maizer and Alhijawi [106] demonstrated the first DL-based progression tracking system using volumetric change analysis across multiple time points, a capability essential for treatment response assessment but absent from 99% of reviewed studies.

### 4.6. Beyond Structural Analysis: Emerging Paradigms

Four emerging paradigms deserve particular attention for their potential to fundamentally reshape brain tumor analysis:

Hemodynamic-informed segmentation. Tang et al. [46] pioneered the integration of DSC perfusion MRI hemodynamic time-course data into deep-learning-based segmentation, using U-Net for structural delineation coupled with HDBScan density-based clustering of perfusion signatures. This approach captures intratumoral hemodynamic heterogeneity—reflecting neovascularization, blood–brain barrier permeability, and metabolic activity—that purely structural T1/T2/FLAIR analysis cannot access. The demonstrated correlation between hemodynamic-informed segmentation volumes and Karnofsky performance status (r = −0.309, not observed with structural-only segmentation) provides prognostically relevant information for treatment planning.

Data security for clinical AI. Mathivanan et al. [101] introduced Secure-Net for encrypted data transmission alongside brain tumor classification, achieving competitive accuracy (95.3–99.7% across three datasets) while addressing HIPAA compliance and institutional data governance requirements. As federated learning matures, notwithstanding the retraction of the study cited in [34], the intersection of model performance, data privacy, and regulatory compliance will become a critical dimension of clinical AI deployment that the research community has only begun to address.

Domain-shift mitigation. Although federated learning may help with privacy issues related to multi-institutional training, it cannot help with addressing scanner, protocol, geography or tumor-type shift. Current strategies for mitigation must be employed in combination to address the various types of shifts: intensity harmonization and scanner-aware data augmentation for acquisition variability, adversarial or feature alignment domain adaptation for inter-site shifts, test-time adaptation for site of deployment shifts, and continual learning for shifts due to the introduction of new scanners and populations. While clinically attractive, continual learning must include drift monitoring, locked external validation sets, and safeguards against catastrophic forgetting.

Generative AI and tumor-progression staging. Sultan et al. [61] introduced what is, to our knowledge, the first integrated DL plus large language model (LLM) clinical pipeline for brain tumor analysis. Their framework first classifies MRI scans into early-stage (EBT) and progressive-stage (PBT) brain tumors using a dedicated classification network with an enhanced-pooled attention block and a dilated fusion block (Acc 85.06%); stage-specific segmentation networks (S1-Net for EBT, S2-Net for PBT) then exploit hierarchical feature fusion to delineate the lesion (DSC 79.96%/81.14%); finally, a clinician-validated GenAI module reads the segmentation masks and patient metadata to produce semantic descriptions of tumor morphology while explicitly reporting interpretive limitations. This represents a methodological shift from classification/segmentation as endpoints to a language-mediated reasoning layer that can communicate findings, uncertainties, and clinically relevant context. In parallel, Sasikala and Anand [43] introduced ExU-Trans, a self-explanatory transformer with a U-Net hybrid that embeds a Discriminative Attribute Explainer (DAE) inside the encoder—producing pixel-level interpretable attributions during segmentation rather than as a post hoc overlay. Together with hybrid attentive autoencoders such as HCAC [47], these designs anticipate the next clinical bottleneck: not raw segmentation accuracy but trustworthy, explainable, language-aware decision support that integrates with radiology workflows.

Capsule routing and ConvNeXt-style hybrids. Two 2026 architectural lines suggest that capacity gains beyond the 2021 transformer plateau may come from rethinking the convolution–attention coupling itself. Kumar and Singh’s HCAC [47] replaces the conventional softmax classifier with a Dwarf-attention Capsule Routing Autoencoder, exploiting capsule networks’ equivariance to spatial transformations to recover the part–whole hierarchies that brain tumor MRI exhibits across slices. Fırat and Üzen’s Brain-CNXSAMNet [49] fuses ConvNeXt’s enlarged receptive fields with a spatial attention module, reaching 99.39% on the consolidated BSF dataset (7023 images) and 98.86% on Figshare in fewer epochs than the strongest baselines—consistent with the hypothesis that ConvNeXt-derived inductive biases compete favorably with full self-attention for medical-image classification at moderate dataset scales.

Foundation models and multi-modal self-supervision. Brain tumor MRI may benefit from self-supervised radiology foundation models trained on large-scale unlabeled MRI volumes, pathology foundation models trained on whole-slide histology, and multi-modal models that align MRI, segmentation masks, molecular markers, operative/pathology reports and radiology text. Their potential role is not only higher Dice but also lower annotation burden, faster adaptation to rare tumors, and interactive clinician prompting. However, these models introduce new validation requirements: pretraining-corpus transparency, prompt sensitivity analysis, calibration under domain shift, and prospective assessment of clinician–AI interaction.

### 4.7. Explainability Methods’ Comparison and Adoption

**Figure 14:**
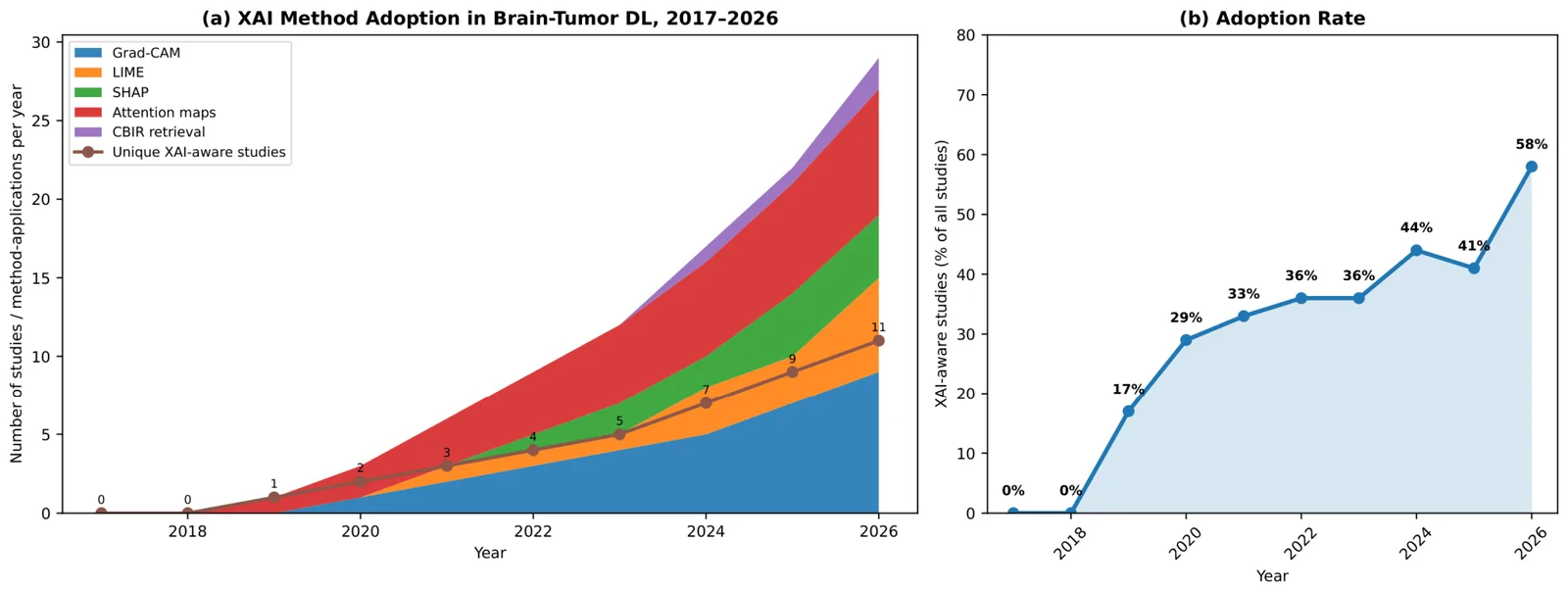
Adoption of explainable AI methods in brain tumor DL studies (2017–2026). Panel (**a**) shows the per-year count of studies using Grad-CAM, LIME, SHAP, attention-map visualization and CBIR retrieval; the black line denotes the total number of XAI-aware studies. Panel (**b**) shows the corresponding adoption rate as a percentage of all studies that year.

The strong push toward clinical readiness has driven sustained growth in the use of explainable AI (XAI) techniques in our corpus, although adoption remains uneven and prospective evaluation of explanations is still rare. Figure 14a summarizes the absolute counts of studies employing each major XAI technique per year between 2017 and 2026; panel (b) renders the corresponding adoption rate. Three trends are clear: (i) Grad-CAM became the de facto baseline (used in 9 of 19 studies in 2026), valued for its simplicity and post hoc applicability to any CNN backbone—HCAC [47], Mazhar et al. [89], Gupta et al. [86], and Gomes and Barbosa [63] all rely on it as a primary saliency tool; (ii) LIME and SHAP have grown more slowly because they require careful sampling and Shapley-value approximation respectively, but their use is now routinely combined with Grad-CAM (e.g., HCAC’s triple-XAI ablation in Table 9 of Kumar and Singh [47] and the Grad-CAM/LIME/Occlusion comparison of Gomes and Barbosa [63]) to triangulate model focus; (iii) content-based image retrieval (CBIR) and self-explanatory architectures—exemplified by Haroun et al. [94] and ExU-Trans [43], respectively—are still rare (2–3 studies in our corpus) but are increasingly cited as the more clinically intuitive direction because they expose either similar prior cases or pixel-level attributions tied to tumor-specific attributes. The XAI adoption rate has risen substantially over the review period and reached roughly 58% in 2026; however, adoption frequency alone does not establish clinical readiness.

### 4.8. Future Research Directions

Based on the empirical evidence synthesized in this review and the critical gaps identified in Section 4.1, Section 4.2, Section 4.3, Section 4.4, Section 4.5 and Section 4.6, ten priority research directions can be articulated. The most pressing methodological priority is prospective multi-site validation: the field must move beyond retrospective evaluations to multi-center clinical trials assessing real-world accuracy, workflow impact, and patient outcomes under IRB/ethics approval, following the template established by Boyd et al. [45] of blinded clinician evaluation with Turing tests and acceptability scoring alongside standard DSC metrics. Closely tied to this is the development of standardized classification benchmarks—public, multi-institutional datasets with expert-consensus labels and molecular annotations for grading, analogous to BraTS for segmentation—because the current reliance on Figshare and Kaggle (single institutions, 2D, limited annotation) fundamentally limits the field’s ability to assess true model performance.

A complementary equity-oriented priority is the expansion of training corpora to under-represented populations, low-resource imaging environments, and diverse scanner manufacturers, building on the BraTS-Africa initiative; the 3–5% DSC degradation observed for under-represented geographies quantifies the equity cost of homogeneous training data. Parallel architectural and infrastructure priorities include foundation models for brain MRI—pretraining large-scale radiology and vision–language models on diverse brain MRI corpora, evaluating parameter-efficient fine-tuning (LoRA, adapters), and extending SAM/MedSAM [35,36] to 3D volumetric brain tumor segmentation with interactive prompting—and federated learning at scale, extending federated frameworks to consortia of at least ten institutions with heterogeneous protocols and integrating data-security mechanisms to address HIPAA/GDPR compliance.

For reproducibility, future benchmark papers should release code, trained weights, Docker/Singularity containers, exact train/validation/test split files, preprocessing scripts, random seeds, hyperparameter schedules, model checkpoints, inference-time scripts and a minimal executable example that reproduces the reported DSC/accuracy table. These items would convert reproducibility from a general aspiration into a concrete checklist that reviewers and readers can verify.

Three further priorities target clinically actionable behavior. Uncertainty-aware segmentation requires integrating calibrated uncertainty maps (Monte Carlo dropout, deep ensembles, evidential deep learning) into clinical workflows for flagging low-confidence regions, with Schwehr and Achanta’s [77] energy-based uncertainty estimation (DSC 0.908 with voxel-level confidence) offering one possible template. Multi-task learning with molecular integration should jointly optimize segmentation, WHO grading, molecular subtyping (IDH, MGMT) and overall survival prediction in unified architectures; combining structural, perfusion and diffusion MRI may be valuable because the 0.66–0.94 AUC range for molecular prediction indicates variable performance from structural MRI alone. Lightweight deployment architectures (knowledge-distilled and pruned models below approximately 20 M parameters) are also relevant for edge deployment and intraoperative use; Ghost-module designs [57] and lightweight hybrids illustrate that substantial parameter reduction can coexist with high reported benchmark accuracy.

Finally, two physiologically and temporally oriented priorities round out the agenda: hemodynamic and functional MRI integration, extending segmentation beyond structural sequences to incorporate perfusion, diffusion, and spectroscopy data [46] so as to capture tumor physiology and treatment response, and longitudinal monitoring and progression tracking, developing and validating DL systems for temporal tumor volume tracking across serial MRI examinations [106] to enable automated RANO application and early recurrence detection.

## 5. Conclusions

This PRISMA-compliant systematic review synthesized 140 studies published between 2017 and 2026 on deep learning for brain tumor segmentation and grading from MRI, including 39 studies in the BraTS Dice-based quantitative comparative analysis. Several converging messages emerge from the synthesis.

For segmentation, the U-Net family, and nnU-Net in particular, remain consistently strong descriptive benchmark baselines rather than a controlled gold standard: nnU-Net v2 reports a whole-tumor Dice similarity coefficient of 0.929 on BraTS 2021, while Swin UNETR provides the highest source-verified enhancing-tumor DSC in Table 2 (0.864). Whole-tumor best-reported values have plateaued in the 0.92–0.93 range since 2020. Inter-rater DSC values reported by Menze et al. [4] were obtained under different reference-standard conditions and should not be interpreted as a directly comparable ceiling. State-space models such as U-Mamba and SegMamba report competitive Dice values with lower GPU memory consumption, but their relative advantages require peer-reviewed replication and broader external validation.

For classification and grading, accuracies above 98% are frequently reported on institutional or public benchmarks such as Figshare and Kaggle, while the cross-dataset studies in this review report decreases of approximately 2.5–15 percentage points. Some heterogeneous reports suggest better preservation of performance in architectures with explicit spatial pathways, including skip connections, and several cascaded segmentation-then-classification studies report 4–8 percentage-point gains over their within-study end-to-end comparators [50,55,59]. These patterns are hypothesis-generating rather than causal findings. Molecular marker prediction from MRI alone remains variable, with reported AUC values spanning approximately 0.66–0.94; evaluation of additional imaging sequences and multi-modal inputs therefore remains an important research direction.

The expansion of the BraTS challenge to meningioma, pediatric and sub-Saharan African cohorts is a critical equity milestone. It quantifies a 3–5% Dice loss for under-represented geographies and exposes the pediatric data-scarcity problem; stepwise transfer learning [45] offers a practical mitigation by reusing adult glioma representations for rare pediatric subtypes. Our QUADAS-AI assessment further reveals that approximately 20% of studies carry a high risk of data-selection bias and 14% lack adequate reference-standard quality; the narrative reporting-bias appraisal in Section 2.9 indicates a likely over-representation of high-performing results across the corpus.

Several emerging paradigms extend DL beyond purely structural analysis toward clinically actionable decision support. These include hemodynamic-informed segmentation [46], contrast-free classification [54], longitudinal progression tracking [106], dual-path explainable frameworks combining classification with content-based image retrieval [94], ConvNeXt with spatial attention [49], capsule-routing autoencoders coupled with triple-XAI [47], self-explanatory transformer encoders that produce interpretable attributions during segmentation [43], and integrated DL plus large-language-model pipelines for tumor-progression staging [61]. Together they shift the field’s focus from raw accuracy toward physiologically informed, longitudinally aware and trustworthy decision support.

A measurable lightweight pivot is also visible in the 2024–2026 cohort. Parameter-efficient designs such as Ghost-ResNet (8.5 M), CerevianNet (6.2 M; 4.1× fewer parameters than its comparator) and FCDS-DeepVision (4.8 M) [57,64] report high benchmark accuracies alongside substantially smaller parameter counts than several legacy VGG and ResNet baselines. This pattern indicates growing interest in deployment-aware design, but the studies are not sufficiently homogeneous for a controlled efficiency ranking. In the 117-study primary corpus, only 15 studies (13%) report independent external or cross-dataset validation; within the 79-study quantitative subset, 12 (15%) report results on two or more independent datasets. Only 23% include any form of explainability (rising to roughly 58% in 2026), and the clinical-translation indicator profile in Figure 13 shows that only 6% provide source code. External validation, interpretability and prospective evaluation should therefore accompany any clinical-deployment claim rather than be treated as optional extensions.

Clinical translation will ultimately require a paradigm shift away from accuracy maximization on convenient benchmarks and toward prospective multi-site validation, calibrated uncertainty quantification, regulatory-pathway engagement, equitable dataset development, and lightweight deployment-ready architectures. The ten research priorities identified in Section 4.8 provide a structured, evidence-grounded roadmap for closing the widening gap between benchmark performance and real-world clinical impact in DL-based brain tumor analysis.

## Figures and Tables

**Table 1:** Database search summary (PRISMA-S compliant). Raw hits are total records returned by the database with the conceptual query; “records screened” reflects the predefined relevance-based stopping rule for each database. Full per-database query strings, filters, search dates, hit counts, and stopping rules are provided in Supplementary File S2.

Database	Date Searched	Raw Hits	Records Screened	Sort	Stopping Rule
Google Scholar	21 May 2026	23,400	∼500	Relevance	First 50 result pages
PubMed/MEDLINE	21 May 2026	5349	∼1100	Best Match	First ∼1100 best-match records
IEEE Xplore	21 May 2026	5233	∼1000	Relevance	First ∼1000 relevance-ranked records
Total	—	33,982	∼2600	—	—

**Table 2:** Segmentation performance of major DL architectures on BraTS benchmarks. WT = whole tumor, TC = tumor core, ET = enhancing tumor. HD95 = 95th-percentile Hausdorff distance (mm). The BraTS column denotes the challenge edition evaluated when stated by the source; generic “BraTS” entries indicate that the edition was not specified. Best-reported values are descriptive upper-envelope values and are not controlled head-to-head rankings.

Method	Family	BraTS	DSC_WT_	DSCTC	DSCET	HD95_WT_	HD95ET
DeepMedic [38]	CNN	2017	0.896	0.832	0.754	6.31	5.42
Myronenko (AE) [39]	U-Net	2018	0.917	0.867	0.823	4.52	3.93
Sun et al. ensemble [40]	CNN	2018	0.909	0.849	0.805	4.80	4.21
DeepSeg [24]	U-Net	2019	0.908	0.845	0.774	5.55	4.89
TransBTS [7]	Trans.	2019	0.911	0.867	0.786	5.24	4.43
nnU-Net v1 [6]	U-Net	2020	0.922	0.892	0.841	4.27	3.61
nnU-Net v2 [6]	U-Net	2021	0.929	0.903	0.854	3.89	3.42
UNETR [8]	Trans.	2021	0.918	0.881	0.840	4.83	3.97
Swin UNETR [9]	Trans.	2021	0.928	0.889	0.864	4.11	3.28
U-Mamba * [10]	SSM	2021	0.921	0.890	0.851	4.25	3.58
SegMamba * [11]	SSM	2021	0.918	0.884	0.845	4.48	3.75
Ali Improved 3D U-Net [41]	U-Net	2018	0.913	0.874	0.801	—	—
Ranjbarzadeh DWA [42]	CNN	2018	0.920	0.873	0.783	—	—
MedNeXt [32]	Hybrid	2023	0.925	0.898	0.858	4.02	3.35
ExU-Trans [43]	Hybrid	BraTS	0.926	0.892	0.860	—	—
Mostafa et al. [37]	U-Net	2020	0.900	—	—	—	—

Rows are restricted to studies with source-verified bibliographic records and directly reported comparable outcomes; the complete study-level decision record is provided in Supplementary File S6. Asterisks identify metrics extracted from non-peer-reviewed preprints, which should be weighted accordingly by readers.

**Table 3:** Performance of DL models for brain tumor classification, grading, and molecular prediction. #Cl = number of output classes, Acc = accuracy (%), Sen = sensitivity (%), Spe = specificity (%), AUC = area under ROC curve. Values are best-reported internal or benchmark results and should not be interpreted as clinical-deployment estimates without patient-level split verification and external validation.

Method	Task	Dataset	#Cl	Acc	Sen	Spe	AUC
Aamir (SE-ResNet + ensemble) [48]	Type class.	Figshare + BraTS	3	99.94	—	—	—
Raza (hybrid DL) [27]	Type class.	Figshare-family	3	99.67	—	—	—
Kumar (HCAC + XAI) [47]	Type class.	Figshare	3	99.90	99.80	—	—
Fırat (Brain-CNXSAMNet) [49]	Type class.	BSF (7023)	4	99.39	—	—	—
Fırat (Brain-CNXSAMNet) [49]	Type class.	Figshare	3	98.86	—	—	—
Sankar (MobileNetV2) [50]	Grade	Multi (6)	2–4	99.85	—	—	—
Abdusalomov (YOLOv7) [30]	Detection	Br35H + Figshare	4	99.50	—	—	—
Ahmad (DLV3 + CNN, ESWA) [51]	Has tumor	BraTS	2	99.31	—	—	—
Gunasekaran (ResNeXt; retracted) [52]	Type class.	BraTS 2020	3	99.27	—	—	—
Jader (Ensemble CNN) [28]	Type class.	BraTS 2020	3	99.16	—	—	—
Tandel (Ensemble 5) [53]	Grade	FLAIR MRI	2	98.88	—	—	—
Lu (Darknet53) [54]	Type class.	In-house (203)	13	98.30	—	—	—
Srinivasan (CRNN) [55]	Grade	REMBRANDT	2	98.17	98.79	91.34	—
Hussain (3D CNN) [56]	Grade	BraTS 2019	2	98.00	—	—	—
Shatnawi (Ghost-ResNet) [57]	Type class.	Bangladesh	3	97.85	97.80	97.80	—
Remzan (RadImageNet) [29]	Type class.	Kaggle MRI	4	97.71	—	—	0.999
Díaz-Pernas (MSCNN) [58]	Type class.	Figshare	3	97.30	94.00	—	—
Guan (ResSGA-Net) [59]	Type class.	Multi (4-cls)	4	98.00	—	—	0.989
Rastogi (Dual-stage) [60]	Has tumor	TCGA-GBM	2	97.00	99.00	—	—
Sultan (DL + GenAI) [61]	EBT vs. PBT	Open prog. ds	2	85.06	85.00	—	—
Srinivas (CNN + XAI) [62]	Type class.	Crystal Clean	4	95.86	—	—	—
Gomes (ViT vs. CNN) [63]	Type class.	Kaggle (7023)	4	98.17	—	—	—
Ali (FCDS-DeepVision) [64]	Type class.	Kaggle (7020)	4	96.50	96.00	—	0.99

Note: Gunasekaran et al. [52] was subsequently retracted by PLOS ONE because of authorship-policy non-compliance, text overlap, unreliable peer review, and citation of a previously retracted source. It is retained only for transparency and historical completeness and is not treated as reliable evidence for comparative or clinical conclusions. Rows are restricted to studies with source-verified bibliographic records and directly reported comparable outcomes; the complete study-level decision record is provided in Supplementary File S6.

**Table 4:** Summary of training configurations across included studies with quantitative data (n = 79). Categories are not mutually exclusive for augmentation.

Category	Strategy	% of Studies
Loss function	Dice + cross-entropy	42%
	Dice loss only	26%
	Cross-entropy only	18%
	Focal loss	8%
	Other (MSE, Log-cosh Dice, PDE)	6%
Optimizer	Adam/AdamW	71%
	SGD (with momentum)	18%
	Other (Adagrad, Lookahead, AdaBelief)	11%
Data augmentation	Random flip/rotation	82%
	Elastic deformation	41%
	Intensity jittering/gamma	37%
	GAN/diffusion synthesis	9%
Normalization	Z-score (zero mean, unit variance)	54%
	Min–max scaling	31%
	Percentile clipping ([1, 99])	15%
Hardware	NVIDIA A100/V100	38%
	NVIDIA RTX 3090/4090	29%
	Other/unreported	33%

**Table 5:** Summary of major brain tumor MRI datasets used in the included studies. mpMRI = T1, T1ce, T2, and FLAIR sequences. #Subj = number of subjects, #Img = total number of images or volumes.

Dataset	Year(s)	#Subj	#Img	Sequences	Annotations	Tumor Types
BraTS 2017–18	2017–18	285	1140	mpMRI	WT, TC, ET	HGG, LGG
BraTS 2019	2019	335	1340	mpMRI	WT, TC, ET	HGG, LGG
BraTS 2020	2020	369	1476	mpMRI	WT, TC, ET	HGG, LGG
BraTS 2021	2021	1251	5004	mpMRI	WT, TC, ET	HGG, LGG
BraTS 2023 Glioma	2023	1470	5880	mpMRI	WT, TC, ET	Adult glioma
BraTS-Africa	2023	60	240	mpMRI	WT, TC, ET	Sub-Saharan
BraTS-PEDs	2023	99	396	mpMRI	WT, TC, ET	Pediatric
BraTS-MenRT	2024	750	3000	mpMRI	GTV	Meningioma
Figshare	2015	3064	3064	T1ce	Outline	Men, Glio, Pit
Kaggle Brain MRI	2020	7023	7023	Mixed	Binary	Mixed
TCGA-GBM/LGG	2016	657	2628	mpMRI	Seg + mol.	GBM, LGG
Br35H	2020	3000	3000	T1ce	Binary	Mixed
REMBRANDT	2005	620	2480	mpMRI	Grade	Glioma

Note: Kaggle/BSF counts are reported as 7023 for the full consolidated image set and 7020 where individual studies used a cleaned sub-set after removing unusable or duplicate images; the denominator is stated where the original study specifies it. Patient/Data selection: 44% low risk, 36% unclear, 20% high risk. High-risk studies typically used cherry-picked subsets of datasets, applied unreported exclusion of difficult cases, or lacked justification for sample selection.

**Table 6:** Representative summary of included primary and contextual studies. Task: Seg = segmentation; Class = classification; Grade = grading/molecular prediction; Det = detection; MT = multi-task; Rev = contextual review. Year denotes publication year, while Dataset(s) reports the evaluated dataset or BraTS edition when stated by the source.

#	Author (Year)	Year	Architecture	Dataset(s)	Task	#Subj	Key Innovation	Best Metric
1	Akkus et al. [14]	2017	Survey	Multiple	Rev	—	First DL survey for brain MRI	—
2	Kamnitsas et al. [38]	2017	DeepMedic	BraTS 2015	Seg	274	Multi-scale 3D CNN + dense CRF	DSC_WT_ 0.896
3	Havaei et al. [65]	2017	Two-path CNN	BraTS 2013	Seg	274	Dual-scale convolutional paths	DSC_WT_ 0.88
4	Myronenko [39]	2019	AE-reg. U-Net	BraTS 2018	Seg	285	Variational AE regularization branch	DSC_WT_ 0.917
5	Sun et al. [40]	2019	Ensemble 3 CNNs	BraTS 2018	Seg	285	Triple-model ensemble + survival head	DSC_WT_ 0.909
6	Zeineldin et al. [24]	2020	DeepSeg	BraTS 2019	Seg	335	Multi-scale FLAIR-focused U-Net	DSC_WT_ 0.908
7	Isensee et al. [6]	2021	nnU-Net	BraTS 2021	Seg	1251	Self-configuring pipeline optimization	DSC_WT_ 0.929
8	Wang et al. [7]	2021	TransBTS	BraTS 2019	Seg	335	First transformer encoder for brain tumor seg	DSC_WT_ 0.911
9	Naser and Deen [66]	2020	U-Net + VGG16	TCGA-LGG/TCIA	MT	110	Joint segmentation + LGG grading	DSC 0.84; patient-level Acc 95%
10	Ranjbarzadeh et al. [42]	2021	Cascade CNN + DWA	BraTS 2018	Seg	285	Cascade with dynamic windowed attention	DSCET 0.911
11	Díaz-Pernas et al. [58]	2021	Multi-scale CNN	Figshare	Class	233	Three-scale hierarchical visual system pathways	Acc 97.3%
12	Raza et al. [27]	2022	Hybrid DL	Figshare	Class	3064	Multi-backbone feature ensemble	Acc 99.67%
13	Arif et al. [67]	2022	U-Net + GA	REMBRANDT	MT	130	GA segmentation + DWT/PSO + U-Net classification	Acc 97%
14	Hatamizadeh et al. [9]	2022	Swin UNETR	BraTS 2021	Seg	1251	Swin Transformer encoder + U-Net decoder	DSCET 0.864
15	Hatamizadeh et al. [8]	2022	UNETR	BraTS 2021	Seg	1251	Pure ViT encoder for volumetric segmentation	DSC_WT_ 0.918
16	Jyothi and Singh [15]	2023	Survey	Multiple	Rev	—	DL + traditional ML comparative review	—
17	Abdusalomov et al. [30]	2023	YOLOv7 + CBAM	Br35H + Figshare	Det	3064	CBAM attention + SPPF + pooling	Acc 99.5%
18	Roy et al. [32]	2023	MedNeXt	BraTS 2023	Seg	1470	ConvNeXt principles for medical 3D seg	DSC_WT_ 0.925
19	Tandel et al. [53]	2023	Ensemble 5 CNN	T1/T2/FLAIR	Grade	620	FLAIR most informative for grading	Acc 98.88%
20	Srinivasan et al. [55]	2023	CRNN	REMBRANDT	Grade	620	CRNN + LBGLCM texture features	Acc 98.17%
21	Soni et al. (retracted) [34]	2023	Fed. multi-enc	BraTS 2018	Seg	285	Federated multi-encoder architecture	DSC_WT_ 0.880
22	Verma and Shivhare [68]	2024	Survey	Multiple	Rev	—	161-article comparative review	—
23	Dorfner et al. [69]	2024	Survey	Multiple	Rev	—	Clinical-focus DL/radiomics review	—
24	Bouhafra and El Bahi [70]	2024	Survey	Multiple	Rev	—	60 articles on DL brain tumor seg	—
25	Jader et al. [28]	2024	Ensemble CNN	BraTS 2020	Class	369	VGG16 + ResNet50 + AlexNet ensemble	Acc 99.16%
26	Ali et al. [41]	2024	Improved 3D U-Net	BraTS 2018	Seg	285	Transition layer + residual blocks	DSC_WT_ 0.913
27	Sankar et al. [50]	2024	MobileNetV2	Multi (6)	Grade	5000+	Six-dataset cross-validation	Acc 99.85%
28	Gunasekaran et al. (retracted) [52]	2024	ResNeXt-101	BraTS 2020	Class	335	AWO feature selection + ConvNet	Acc 99.27%
29	Remzan et al. [29]	2024	RadImageNet	Kaggle MRI	Class	7023	RadImageNet pretrained weights	Acc 97.71%, AUC 0.999
30	Ma et al. [10]	2024	U-Mamba	BraTS 2021	Seg	1251	Mamba state-space encoder, 40% less memory	DSC_WT_ 0.921
31	Xing et al. [11]	2024	SegMamba	BraTS 2021	Seg	1251	Long-range SSM for volumetric seg	DSC_WT_ 0.918
32	Wang et al. [71]	2024	Meta-analysis	Multiple	Rev	12,000	Systematic review/meta-analysis of externally validated AI detection/segmentation	Pooled lesion-wise DSC 0.84
33	Kaur et al. [72]	2025	Survey	Multiple	Rev	—	PRISMA review across 220 articles	—
34	Abid and Munir [73]	2025	Survey	Multiple	Rev	—	80-article DL architecture taxonomy	—
35	Missaoui et al. [74]	2025	Survey	Multiple	Rev	—	107 studies ML + DL comprehensive review	—
36	Islam et al. [75]	2025	CBAM U-Net	BraTS	Seg	—	CBAM attention + VGG19 encoder	—
37	Iqbal et al. [76]	2025	3D ResU-Net	BraTS 2021	MT	1251	Joint seg + MGMT methylation pred	DSC 0.83, AUC 0.66
38	Aamir et al. [48]	2025	SE-ResNet + Ens.	Figshare + BraTS	Class	3064	Multi-scale features + triple attention	Acc 99.94%
39	Lu et al. [54]	2025	Darknet53 + FCN	203 subj + BraTS	MT	203	Non-contrast RGB MRI fusion	Acc 98.3%, DSC 0.937
40	Schwehr and Achanta [77]	2025	Att. U-Net	BraTS 19/20/21	Seg	1251	Energy-based uncertainty estimation	DSC 90.82 (BraTS 2021)
41	Echine and Darouichi [78]	2025	3D AGRes-UNet	BraTS 2020	Seg	369	ASPP + attention gates for 3D seg	DSC_WT_ 0.822
42	Rasool et al. [79]	2025	CNN-TumorNet	Kaggle MRI	Class	—	LIME-based spatial explainability	Acc 99%
43	Ganesh et al. [80]	2025	VGG16 hybrid	BraTS 2020	Class	369	VGG16 + shallow CNN fusion	Acc 97.15%
44	Jin et al. [81]	2025	Survey	Multiple	Rev	—	Semi-supervised DL brain tumor review	—
45	Saifullah et al. [82]	2025	Survey	Multiple	Rev	—	Metaheuristic optimization + DL review	—
46	Bastug and Akgun [83]	2026	Survey	Multiple	Rev	—	U-Net variants PRISMA-S review	—
47	Prasad (CoHeD) [84]	2026	Hybrid CNN-Tr.	Multi-center	MT	—	Consistency-guided dual-task learning	DSC 0.904, Acc 92.6%
48	Ullah (Bayes. DLV3+) [85]	2026	Bayesian hybrid	BraTS 23 + Figsh.	MT	—	PDE-informed loss + Bayesian optimization	DSC 95.86%, Acc 98.8%
49	Gupta (FL-XAI) [86]	2026	Fed. ResNet-18	Kaggle (4 inst.)	Class	—	Privacy-preserving federated + XAI	Acc 92% (FL)
50	Berwer (RP-SegNet) [87]	2026	RP-SegNet	BraTS 20 + Figsh.	Seg	369	Recursive residual + blockchain security	DSC 95.07%
51	Chen (SwitchNet) [88]	2026	U-Net variant	BraTS 23 + ISLES	Seg	1470	Adaptive distribution switching mechanism	DSC_WT_ 0.919
52	Mazhar et al. [89]	2026	EfficientNetB0	Kaggle + Multi	Class	10 k+	Lightweight triple-XAI pipeline	Acc 99.45%
53	Muksimova (CT-UNet) [90]	2026	Transformer U-Net	BraTS	Seg	—	Bidirectional Transformer + DMF fusion	DSC 0.92
54	Gasmi et al. [91]	2026	3D U-Net Pool	BraTS 2020	Seg	369	Fuzzy 3D pooling strategy	DSC_WT_ 0.899
55	Zhou et al. [92]	2026	Teacher-Student	BraTS 18/19/20	Seg	369	Modality-agnostic knowledge distillation	DSC 85.9% (2020)
56	Moshe et al. [93]	2026	DDPM + MI	BraTS + TASMC	Class	559	Diffusion-based synthetic augmentation + MI	Cross-ds Acc 89%
57	Haroun et al. [94]	2026	GhostNetV3	Cheng/Figshare CE-MRI	Class	233 (3064 images)	GhostNetV3 + deformable conv + CBIR retrieval	Acc 99.71%, CRAS 0.96
58	Ye et al. [95]	2026	YOLOv12n	Kaggle	Det	3064	A2C2f module + CGAFusion	mAP@0.5 94.0%
59	Ahmad et al. [51]	2026	DeepLabV3 + CNN	BraTS 2015	MT	—	Bilayer governance architecture	Acc 99.31%, DSC 0.963
60	Jahan et al. [96]	2026	CerevianNet	Multi (5)	Class	—	Lightweight: 4.1× fewer parameters	Acc 98.30%
61	Pasunoori et al. [97]	2026	Survey	Multiple	Rev	—	Past–present–future review	—
62	Farahani et al. [98]	2024	Meta-analysis	Multiple	Rev	—	IDH/1p19q MRI prediction systematic review/meta-analysis	AUC 0.89 (IDH); 0.90 (1p/19q)
63	Ghadimi et al. [99]	2025	Survey	Multiple	Rev	—	Multiparametric MRI + DL glioma segmentation review	—
64	Jiménez-Partinen et al. [44]	2026	Preprocessing comparison	UPenn-GBM + MSLesSeg + epilepsy	Seg	—	Intensity-regularization/preprocessing comparison	≈3–6% DSC variation
65	Adewole et al. [18]	2023	BraTS-Africa	60 subjects	Seg	60	First sub-Saharan African brain tumor benchmark	DSC_WT_ 0.87
66	LaBella et al. [17]	2024	BraTS-MenRT	750 subjects	Seg	750	Meningioma radiotherapy planning benchmark	DSC 0.815
67	Baid et al. [13]	2021	BraTS 2021	1251 subjects	Seg	1251	Largest multi-institutional glioma benchmark	Top DSC_WT_ 0.929
68	Boyd et al. [45]	2024	Stepwise TL	CBTN + DFCI/BCH	Seg	284	Stepwise adult transfer learning	DSC 0.82 (ext.)
69	Tang et al. [46]	2024	U-Net + HDBScan	UPENN-GBM	Seg	513	Hemodynamic perfusion-informed segmentation	IoU 74.8%, KPS corr.
70	Ilani et al. [100]	2025	U-Net (adapted)	Figshare + Kaggle	Class	3064	Cross-dataset generalizability analysis	Acc 96.01% (ext.)
71	Mathivanan et al. [101]	2025	BTDN + Secure-Net	3 datasets	Class	10 k+	Encrypted pipeline for clinical compliance	Acc 95.3–99.7%
72	Rastogi et al. [102]	2025	3D Rep. + Vol. CNN	BraTS 2020	Seg	150	Replicator network + survival prediction	DSC 0.857–0.878
73	Shatnawi et al. [57]	2026	Ghost-ResNet50	Bangladesh (6 k)	Class	6056	Lightweight ECA + ghost modules	Acc 97.85%
74	Hussain et al. [56]	2026	3D U-Net + 3D CNN	BraTS 2019	MT	335	Cascaded seg pipeline	Acc 98%, DSC 0.95
75	Fasihi Shirehjini [103]	2026	Survey	BraTS (145 stud.)	Rev	—	BraTS-focused DL systematic review	—
76	Goyal et al. [104]	2026	Survey	Multi (161 art.)	Rev	—	ViT + XAI brain tumor review	—
77	Bindu and Uma Devi [105]	2026	Optimized DL	BraTS 3D	Class	—	3D MRI brain tumor classification optimization	High accuracy
78	Kumar and Singh (HCAC) [47]	2026	IroLF + MDU + SwinCT + CapsNet	Figshare/Kaggle/GBM	MT	7000+	Hybrid attentive autoencoder + Grad-CAM/LIME/SHAP	Acc 99.9%, IoU 0.996, Dice 0.97
79	Fırat and Üzen [49]	2026	Brain-CNXSAMNet	BSF + Figshare	Class	7023	ConvNeXt + Spatial Attention Module	Acc 99.39% (BSF), 98.86% (Figshare)
80	Guan et al. (ResSGA-Net) [59]	2026	ResNet50 + Swin + dual attention	Multi (4-/3-cls)	Class	7023+	Convolutional + transformer + global/gated attention	Acc 98%, AUC 0.989
81	Sultan et al. (GenAI) [61]	2026	C-Net + S1/S2-Net + LLM	Brain prog. ds	MT	—	Generative AI + stage-specific seg (EBT vs. PBT)	Acc 85.06%, Dice 81.14%
82	Sasikala and Anand (ExU-Trans) [43]	2026	ViT + U-Net hybrid	BraTS	Seg	—	Self-explanatory MHA + DAE pixel-level interpretability	Improved DSC + interpretability
83	Ali et al. (FCDS-DeepVision) [64]	2026	Lightweight CNN	Kaggle (7020)	Class	7020	Built-from-scratch lightweight, real-time inference	Acc 96.5%, AUC 0.99
84	Gomes and Barbosa [63]	2026	9-arch comparison	Kaggle (7023)	Class	7023	ViT vs. CNN + Grad-CAM/LIME/Occlusion sensitivity	Acc 98.17% (ViT-B/16)
85	Srinivas and Parvathi [62]	2026	4-conv-1-dense CNN	Crystal Clean MRI	Class	—	Compact CNN beats deeper TL models with XAI	Acc 95.86%
86	Rastogi et al. (dual-stage) [60]	2026	MobileNet/NASNet/ResNet101 + RESUNET	TCGA-GBM	MT	3929	Cascaded transfer-learning classification + segmentation	Acc 97%, Recall 99%

Note: Gunasekaran et al. [52] was subsequently retracted by PLOS ONE because of authorship-policy non-compliance, text overlap, unreliable peer review, and citation of a previously retracted source; it is retained only for transparency and historical completeness and is not treated as reliable evidence. Soni et al. [34] was also subsequently retracted after a compromised peer-review process and missing required study-approval and patient-consent statements; the editors considered its results unreliable. It is likewise retained only for transparency and is not treated as reliable evidence.

## Data Availability

All data analyzed in this review are available in the respective published articles cited herein. The review protocol is preregistered on the Open Science Framework (OSF; DOI 10.17605/OSF.IO/C2QA6, https://osf.io/c2qa6 (accessed on 30 July 2026)). The PRISMA 2020 checklist, PRISMA-S search-strategy report, master data extraction sheet, QUADAS-AI domain summary, contextual-reviews list, evidence-locked study decision log, figure source data, and reproducible figure-generation script are provided as Supplementary Files S1–7 and are also available from the corresponding author upon reasonable request.

## Supplementary Materials

The following supporting information can be downloaded at: https://www.mdpi.com/article/10.3390/diagnostics16172806/s1, The following Supplementary Files Saccompany this manuscript and are also available via the OSF project (https://osf.io/c2qa6 (accessed on 30 July 2026)): Supplementary File S1: Completed PRISMA 2020 checklist. Supplementary File S2: PRISMA-S search-strategy report with per-database queries, filters, dates, raw hits, and stopping rules. Supplementary File S3: Master data extraction database (CSV) with all extracted fields for the 117 primary studies. Supplementary File S4: QUADAS-AI domain-level risk-of-bias summary, appraisal criteria, and interpretation for the 117 primary studies. Supplementary File S5: Contextual reviews list (23 entries) with summary fields. Supplementary File S6: Evidence-locked study decision log for the 141 full-text records assessed (140 included and one excluded). Supplementary File S7: Figure source data, PRISMA flow counts, and the reproducible figure-generation script.

## Abbreviations

AUC: Area Under the Receiver Operating Characteristic Curve; BraTS: Brain Tumor Segmentation Challenge; CBAM: Convolutional Block Attention Module; CBIR: Content-Based Image Retrieval; CE: Contrast-Enhanced; CNN: Convolutional Neural Network; CNS: Central Nervous System; DDPM: Denoising Diffusion Probabilistic Model; DL: Deep Learning; DSC: Dice Similarity Coefficient; ET: Enhancing Tumor; FLAIR: Fluid-Attenuated Inversion Recovery; GAN: Generative Adversarial Network; GBM: Glioblastoma Multiforme; GTV: Gross Tumor Volume; HD95: 95th-Percentile Hausdorff Distance; HGG: High-Grade Glioma; IDH: Isocitrate Dehydrogenase; LGG: Low-Grade Glioma; MGMT: O6-Methylguanine-DNA Methyltransferase; mpMRI: Multi-Parametric Magnetic Resonance Imaging; MRI: Magnetic Resonance Imaging; PRISMA: Preferred Reporting Items for Systematic Reviews and Meta-Analyses; PRISMA-S: PRISMA Search-Strategy Reporting Extension; QUADAS-2: Quality Assessment of Diagnostic Accuracy Studies-2; QUADAS-AI: AI-adapted QUADAS framework used in this review; OSF: Open Science Framework; RQ: Research Question; SAM: Segment Anything Model; SLR: Systematic Literature Review; SSM: State-Space Model; TC: Tumor Core; WHO: World Health Organization; WT: Whole Tumor; XAI: Explainable Artificial Intelligence.
