# Supplementary material for: Brain Tumor Segmentation and Grading on MRI Using Deep Learning: A Systematic Literature Review and Benchmark-Driven Comparative Analysis
Source: Diagnostics (Basel). 2026 Aug 31;16(17):2806. doi: 10.3390/diagnostics16172806 (PMC13565208; doi:10.3390/diagnostics16172806)
Supplement: Supplementary file 1 [file diagnostics-16-02806-s001.zip › Supplementary File S7_Figure_Data_and_Reproducibility.zip!/rendered_figures/architecture_dataset_distribution.pdf]

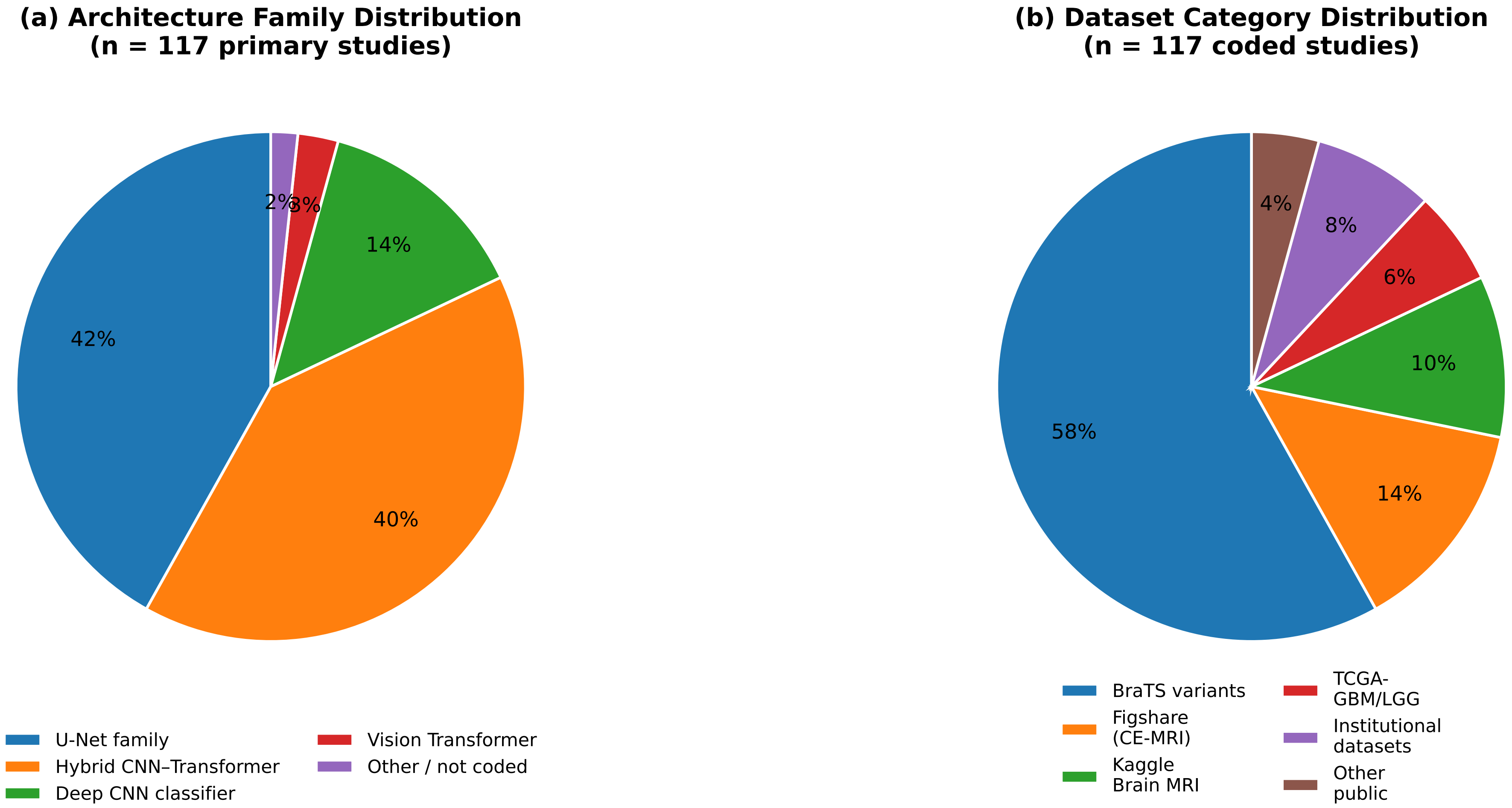
(a) Architecture Family Distribution
(n = 117 primary studies)
2%
3%
14%
42%
40%
U-Net family
Hybrid CNN–Transformer
Deep CNN classifier
Vision Transformer
Other / not coded
(b) Dataset Category Distribution
(n = 117 coded studies)
4%
8%
6%
10%
58%
14%
BraTS variants
Figshare (CE-MRI)
Kaggle Brain MRI
TCGA-GBM/LGG
Institutional datasets
Other public
