# Supplementary material for: Brain Tumor Segmentation and Grading on MRI Using Deep Learning: A Systematic Literature Review and Benchmark-Driven Comparative Analysis
Source: Diagnostics (Basel). 2026 Aug 31;16(17):2806. doi: 10.3390/diagnostics16172806 (PMC13565208; doi:10.3390/diagnostics16172806)
Supplement: Supplementary file 1 [file diagnostics-16-02806-s001.zip › Supplementary File S7_Figure_Data_and_Reproducibility.zip!/rendered_figures/clinical_translation_funnel.pdf]

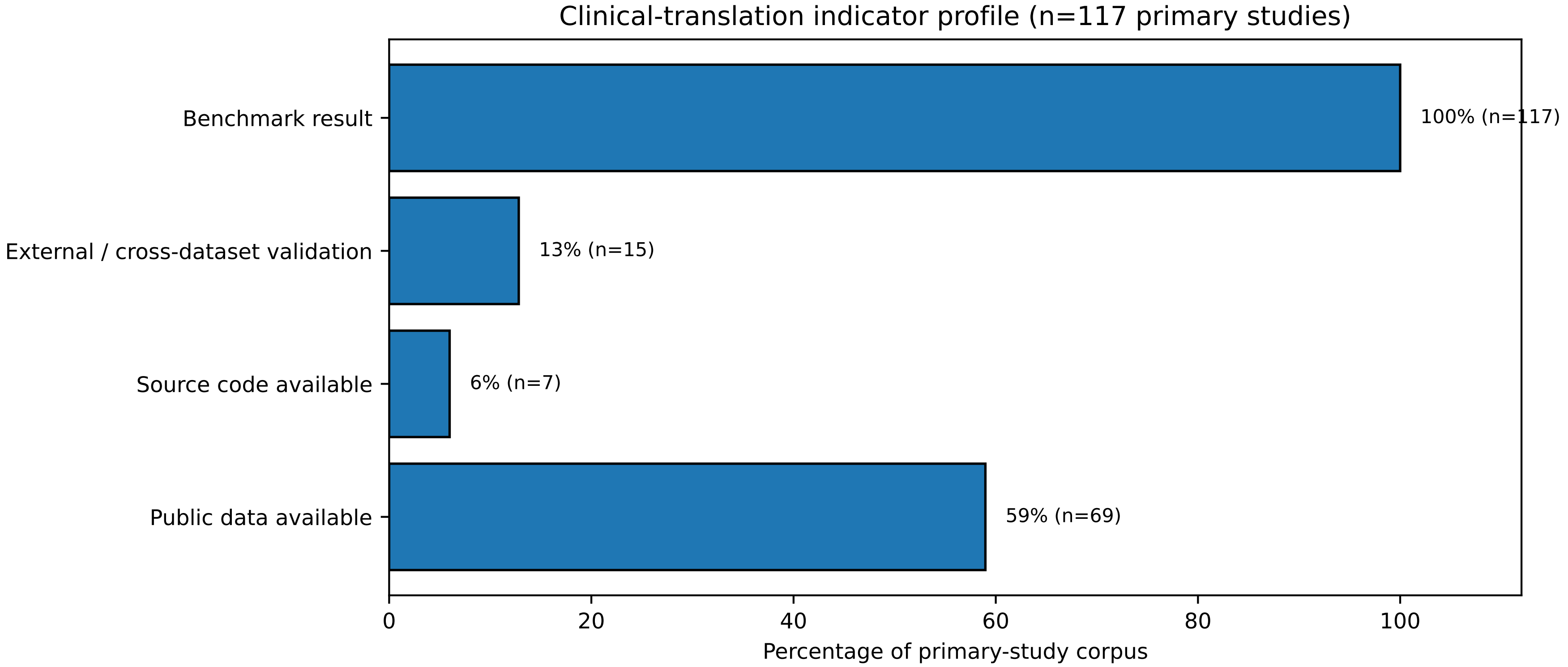
Clinical-translation indicator profile (n=117 primary studies)
Benchmark result
External / cross-dataset validation
Source code available
Public data available
100% (n=117)
13% (n=15)
6% (n=7)
59% (n=69)
0
20
40
60
80
100
Percentage of primary-study corpus
