# Supplementary material for: Brain Tumor Segmentation and Grading on MRI Using Deep Learning: A Systematic Literature Review and Benchmark-Driven Comparative Analysis
Source: Diagnostics (Basel). 2026 Aug 31;16(17):2806. doi: 10.3390/diagnostics16172806 (PMC13565208; doi:10.3390/diagnostics16172806)
Supplement: Supplementary file 1 [file diagnostics-16-02806-s001.zip › Supplementary File S7_Figure_Data_and_Reproducibility.zip!/rendered_figures/dsc_distribution.pdf]

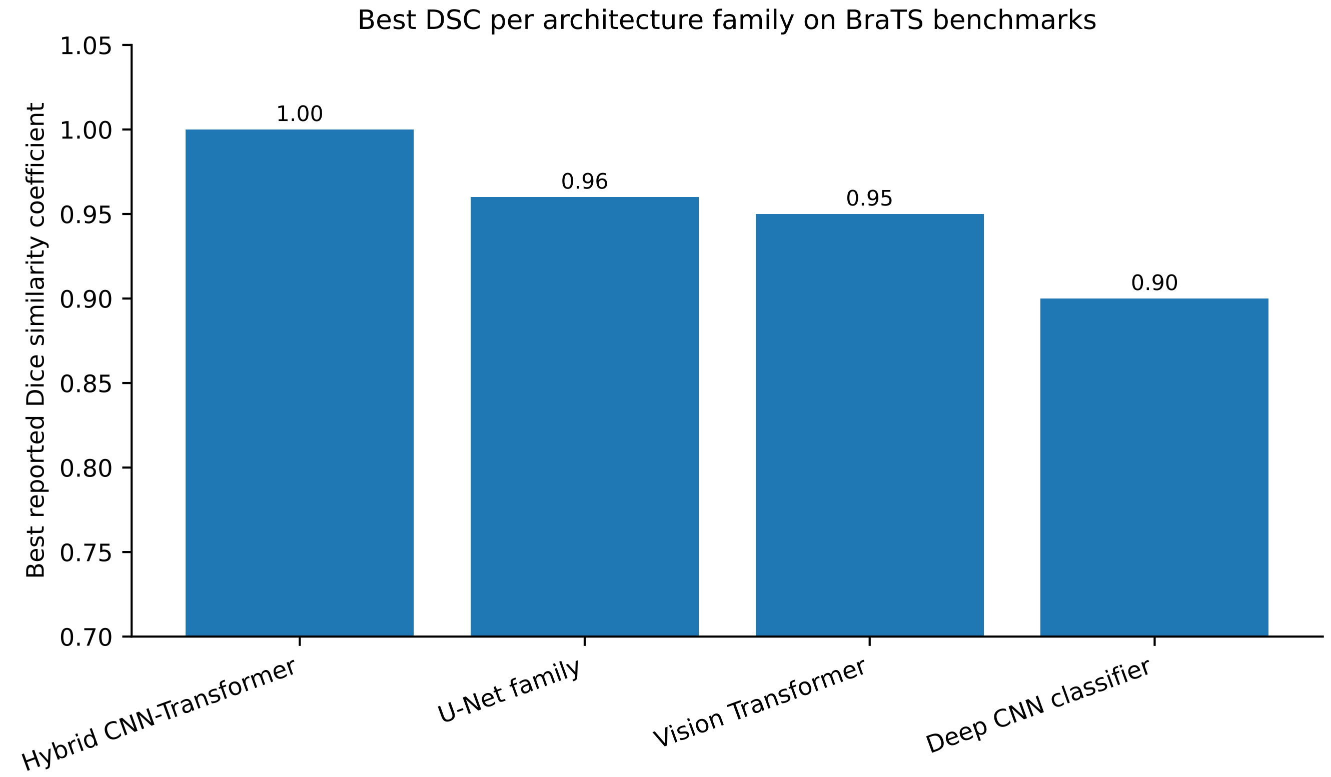

Best DSC per architecture family on BraTS benchmarks
Best reported Dice similarity coefficient
1.05
1.00
0.95
0.90
0.85
0.80
0.75
0.70
1.00
0.96
0.95
0.90
Hybrid CNN-Transformer
U-Net family
Vision Transformer
Deep CNN classifier
