# Supplementary material for: Brain Tumor Segmentation and Grading on MRI Using Deep Learning: A Systematic Literature Review and Benchmark-Driven Comparative Analysis
Source: Diagnostics (Basel). 2026 Aug 31;16(17):2806. doi: 10.3390/diagnostics16172806 (PMC13565208; doi:10.3390/diagnostics16172806)
Supplement: Supplementary file 1 [file diagnostics-16-02806-s001.zip › Supplementary File S7_Figure_Data_and_Reproducibility.zip!/rendered_figures/dsc_trend.pdf]

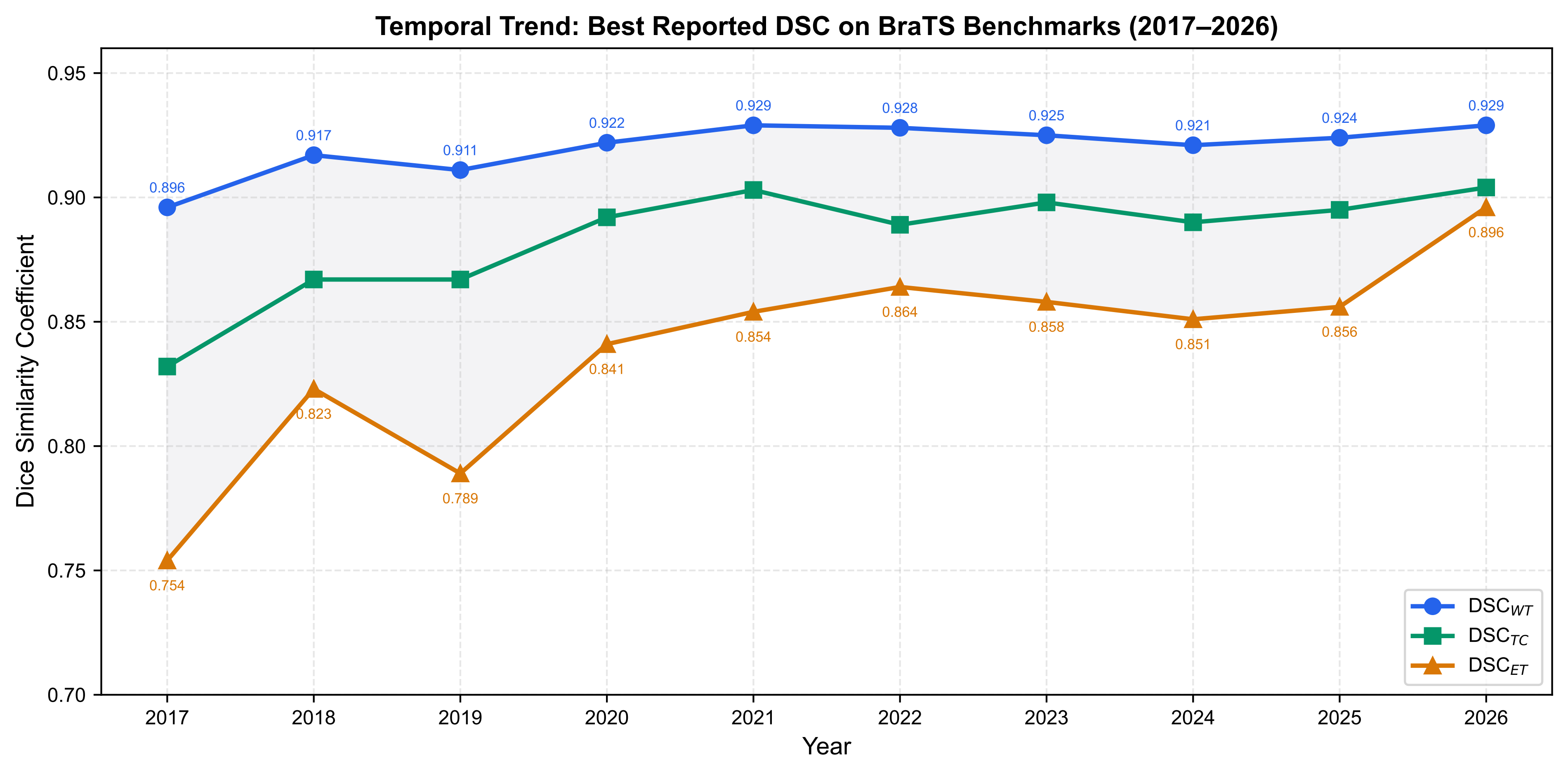

Temporal Trend: Best Reported DSC on BraTS Benchmarks (2017–2026)
Dice Similarity Coefficient
Year
0.70
0.75
0.80
0.85
0.90
0.95
2017
2018
2019
2020
2021
2022
2023
2024
2025
2026
0.896
0.917
0.911
0.922
0.929
0.928
0.925
0.921
0.924
0.929
0.754
0.823
0.789
0.841
0.854
0.864
0.858
0.851
0.856
0.896
DSC_{WT}
DSC_{TC}
DSC_{ET}
