# Supplementary material for: Brain Tumor Segmentation and Grading on MRI Using Deep Learning: A Systematic Literature Review and Benchmark-Driven Comparative Analysis
Source: Diagnostics (Basel). 2026 Aug 31;16(17):2806. doi: 10.3390/diagnostics16172806 (PMC13565208; doi:10.3390/diagnostics16172806)
Supplement: Supplementary file 1 [file diagnostics-16-02806-s001.zip › Supplementary File S7_Figure_Data_and_Reproducibility.zip!/rendered_figures/efficiency_tradeoff.pdf]

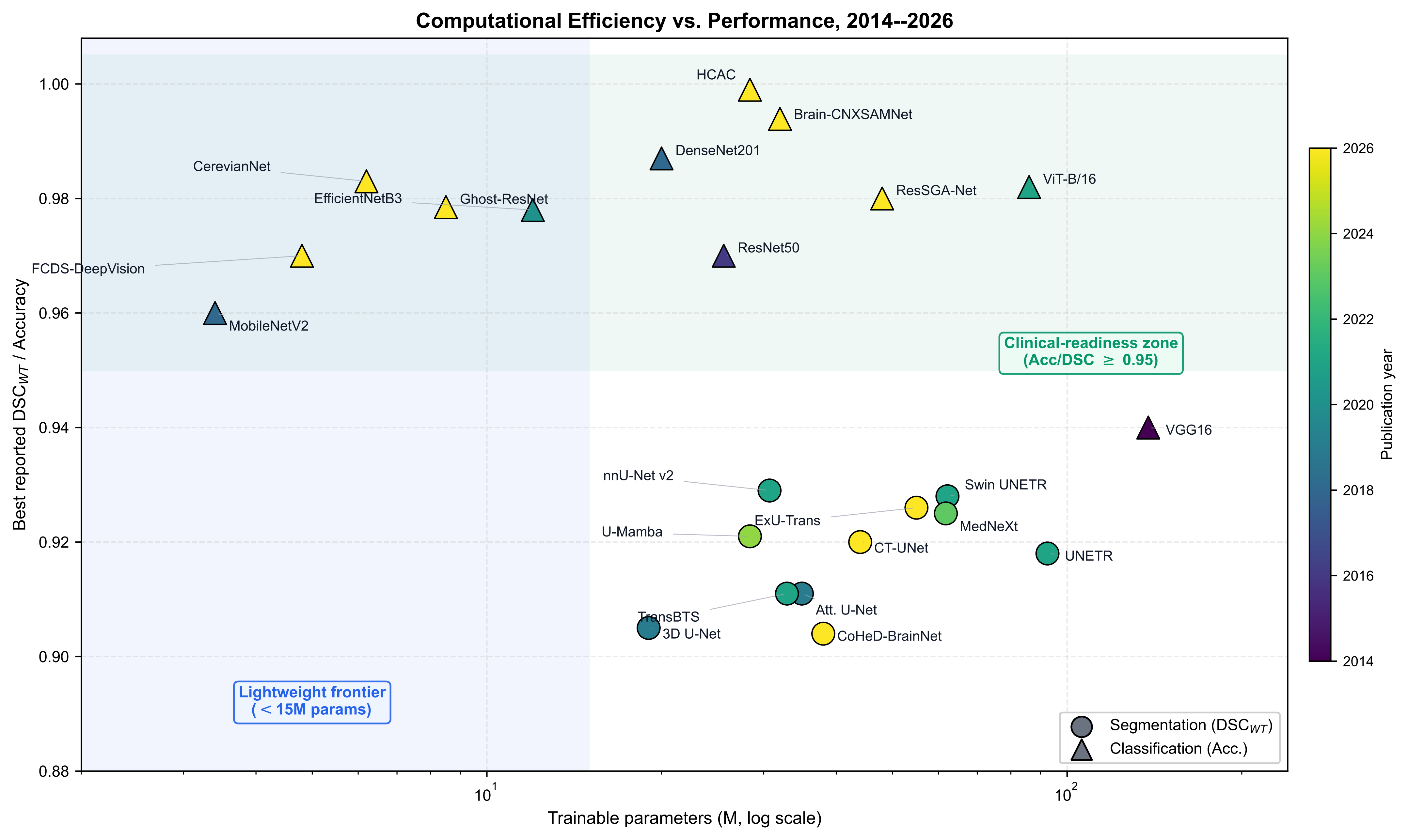

Computational Efficiency vs. Performance, 2014--2026
Best reported DSC$_{WT}$ / Accuracy
Trainable parameters (M, log scale)
Publication year
HCAC
Brain-CNXSAMNet
DenseNet201
CerevianNet
EfficientNetB3
Ghost-ResNet
ViT-B/16
ResSGA-Net
ResNet50
FCDS-DeepVision
MobileNetV2
Clinical-readiness zone
(Acc/DSC ≥ 0.95)
VGG16
nnU-Net v2
Swin UNETR
ExU-Trans
MedNeXt
U-Mamba
CT-UNet
UNETR
TransBTS
Att. U-Net
3D U-Net
CoHeD-BrainNet
Lightweight frontier
(< 15M params)
Segmentation (DSC$_{WT}$)
Classification (Acc.)
