# Supplementary material for: Brain Tumor Segmentation and Grading on MRI Using Deep Learning: A Systematic Literature Review and Benchmark-Driven Comparative Analysis
Source: Diagnostics (Basel). 2026 Aug 31;16(17):2806. doi: 10.3390/diagnostics16172806 (PMC13565208; doi:10.3390/diagnostics16172806)
Supplement: Supplementary file 1 [file diagnostics-16-02806-s001.zip › Supplementary File S7_Figure_Data_and_Reproducibility.zip!/rendered_figures/methodology_fingerprint.pdf]

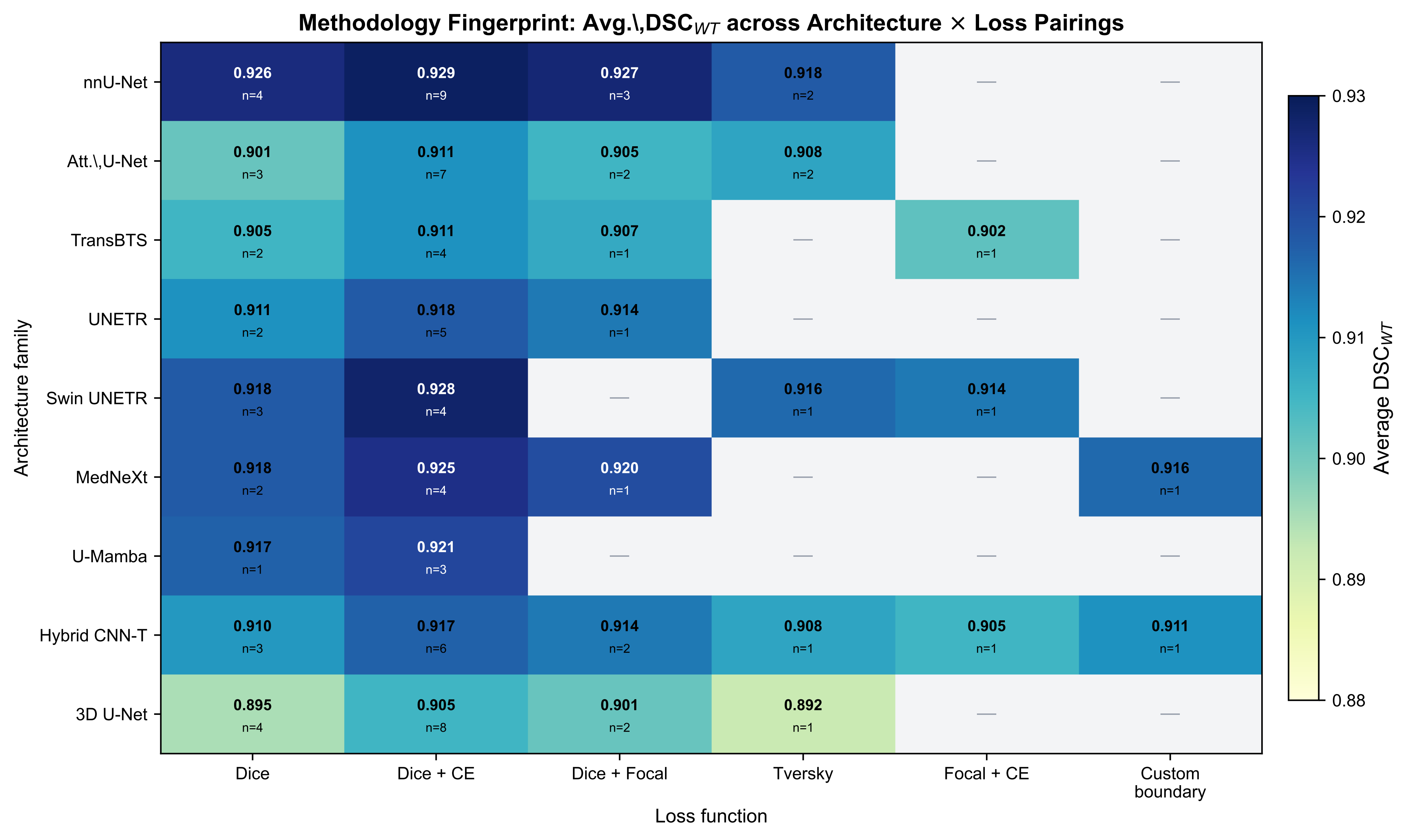
Methodology Fingerprint: Avg.\,DSC$_{WT}$ across Architecture × Loss Pairings
Architecture family
Loss function
Average DSC$_{WT}$
0.93
0.92
0.91
0.90
0.89
0.88
Dice
Dice + CE
Dice + Focal
Tversky
Focal + CE
Custom boundary
nnU-Net
0.926 n=4
0.929 n=9
0.927 n=3
0.918 n=2
Att.\,U-Net
0.901 n=3
0.911 n=7
0.905 n=2
0.908 n=2
TransBTS
0.905 n=2
0.911 n=4
0.907 n=1
0.902 n=1
UNETR
0.911 n=2
0.918 n=5
0.914 n=1
Swin UNETR
0.918 n=3
0.928 n=4
0.916 n=1
0.914 n=1
MedNeXt
0.918 n=2
0.925 n=4
0.920 n=1
0.916 n=1
U-Mamba
0.917 n=1
0.921 n=3
Hybrid CNN-T
0.910 n=3
0.917 n=6
0.914 n=2
0.908 n=1
0.905 n=1
0.911 n=1
3D U-Net
0.895 n=4
0.905 n=8
0.901 n=2
0.892 n=1
