# Supplementary material for: Brain Tumor Segmentation and Grading on MRI Using Deep Learning: A Systematic Literature Review and Benchmark-Driven Comparative Analysis
Source: Diagnostics (Basel). 2026 Aug 31;16(17):2806. doi: 10.3390/diagnostics16172806 (PMC13565208; doi:10.3390/diagnostics16172806)
Supplement: Supplementary file 1 [file diagnostics-16-02806-s001.zip › Supplementary File S7_Figure_Data_and_Reproducibility.zip!/rendered_figures/performance_heatmap.pdf]

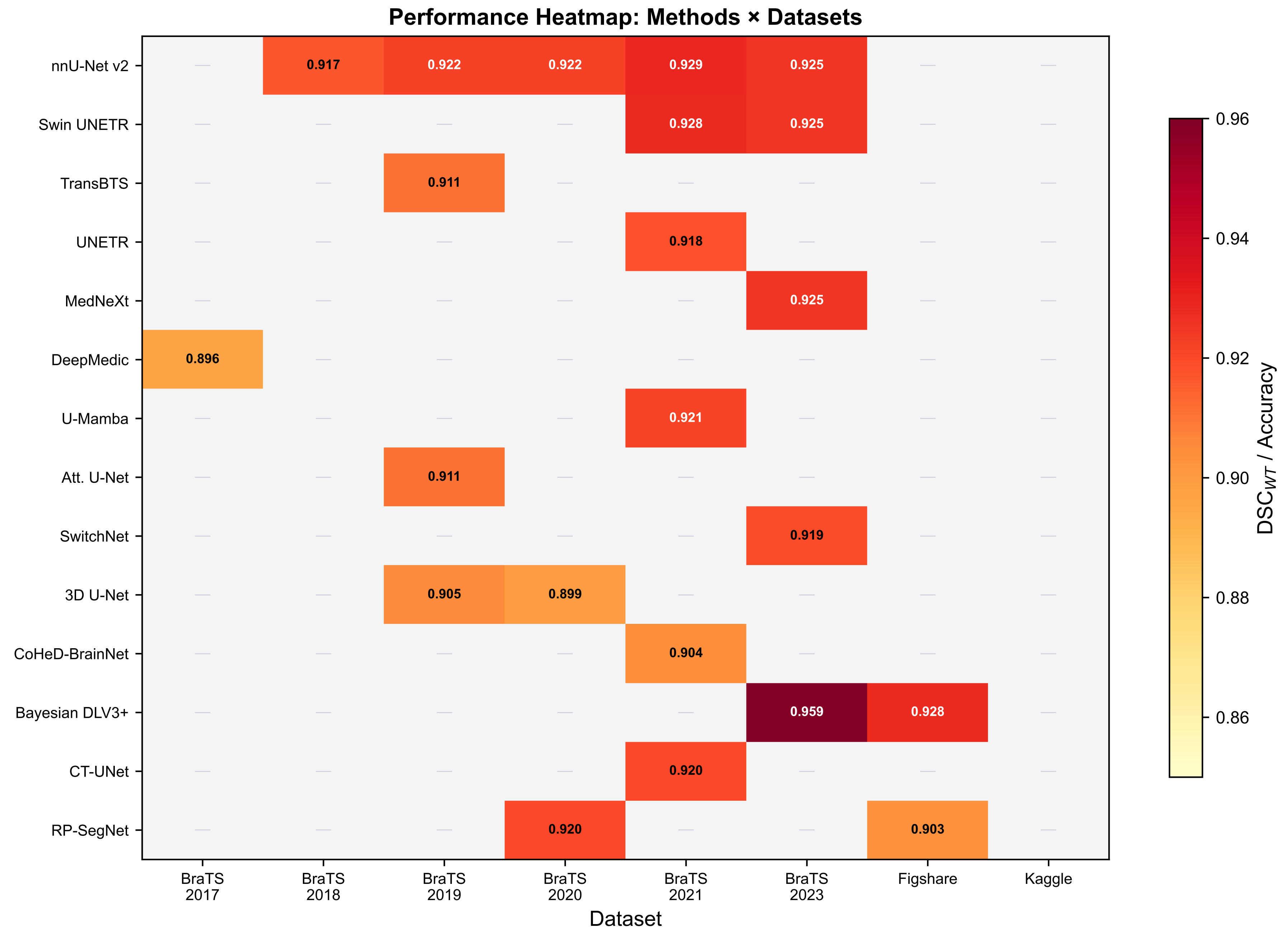

Performance Heatmap: Methods × Datasets
nnU-Net v2
Swin UNETR
TransBTS
UNETR
MedNeXt
DeepMedic
U-Mamba
Att. U-Net
SwitchNet
3D U-Net
CoHeD-BrainNet
Bayesian DLV3+
CT-UNet
RP-SegNet
0.917
0.922
0.922
0.929
0.925
0.928
0.925
0.911
0.918
0.925
0.896
0.921
0.911
0.919
0.905
0.899
0.904
0.959
0.928
0.920
0.920
0.903
BraTS 2017
BraTS 2018
BraTS 2019
BraTS 2020
BraTS 2021
BraTS 2023
Figshare
Kaggle
Dataset
0.96
0.94
0.92
0.90
0.88
0.86
DSC_{WT} / Accuracy
