# Supplementary material for: Brain Tumor Segmentation and Grading on MRI Using Deep Learning: A Systematic Literature Review and Benchmark-Driven Comparative Analysis
Source: Diagnostics (Basel). 2026 Aug 31;16(17):2806. doi: 10.3390/diagnostics16172806 (PMC13565208; doi:10.3390/diagnostics16172806)
Supplement: Supplementary file 1 [file diagnostics-16-02806-s001.zip › Supplementary File S7_Figure_Data_and_Reproducibility.zip!/rendered_figures/quality_assessment.pdf]

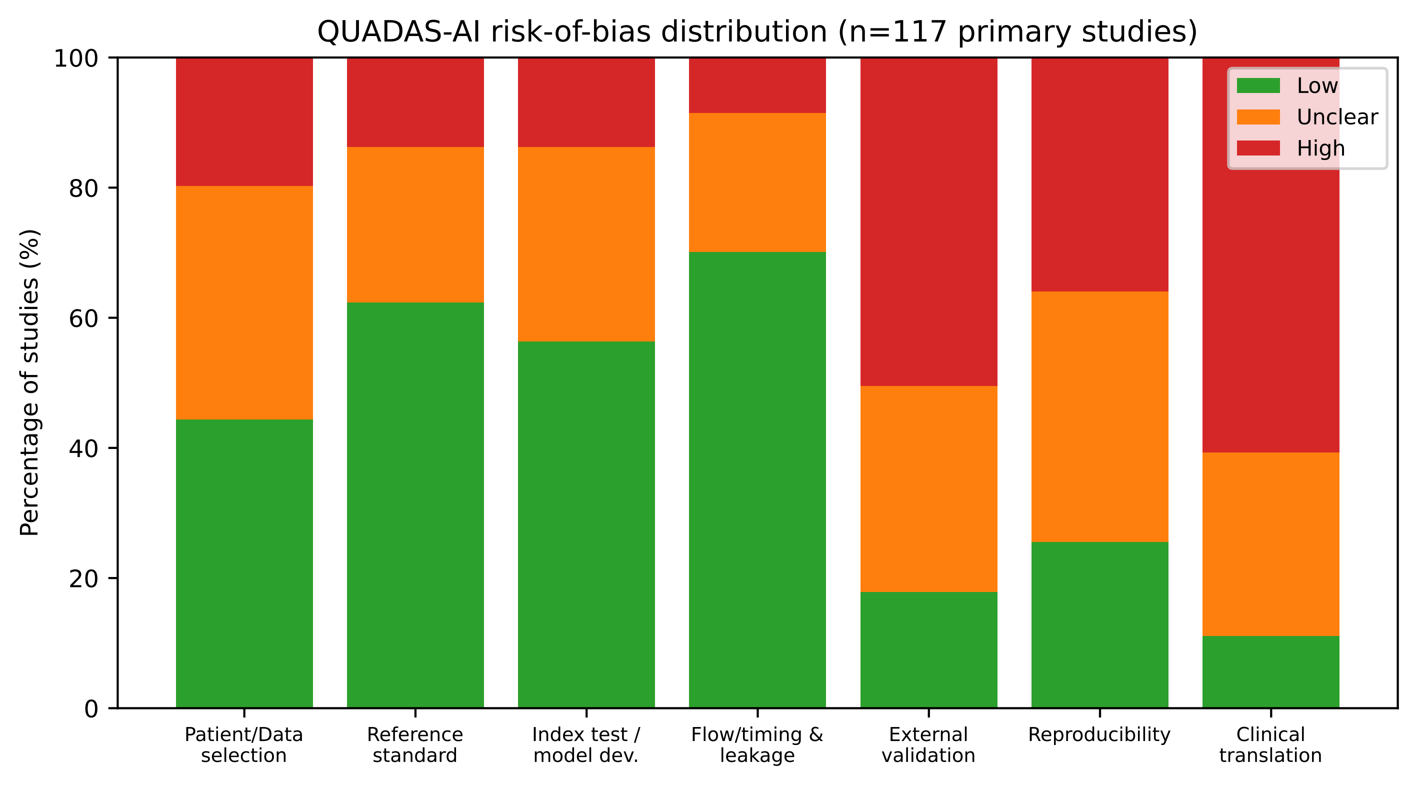
QUADAS-AI risk-of-bias distribution (n=117 primary studies)
Percentage of studies (%)
0
20
40
60
80
100
Low
Unclear
High
Patient/Data selection
Reference standard
Index test / model dev.
Flow/timing & leakage
External validation
Reproducibility
Clinical translation
