# Supplementary material for: Brain Tumor Segmentation and Grading on MRI Using Deep Learning: A Systematic Literature Review and Benchmark-Driven Comparative Analysis
Source: Diagnostics (Basel). 2026 Aug 31;16(17):2806. doi: 10.3390/diagnostics16172806 (PMC13565208; doi:10.3390/diagnostics16172806)

Multi-Dimensional Comparison of Top-5 Architectures

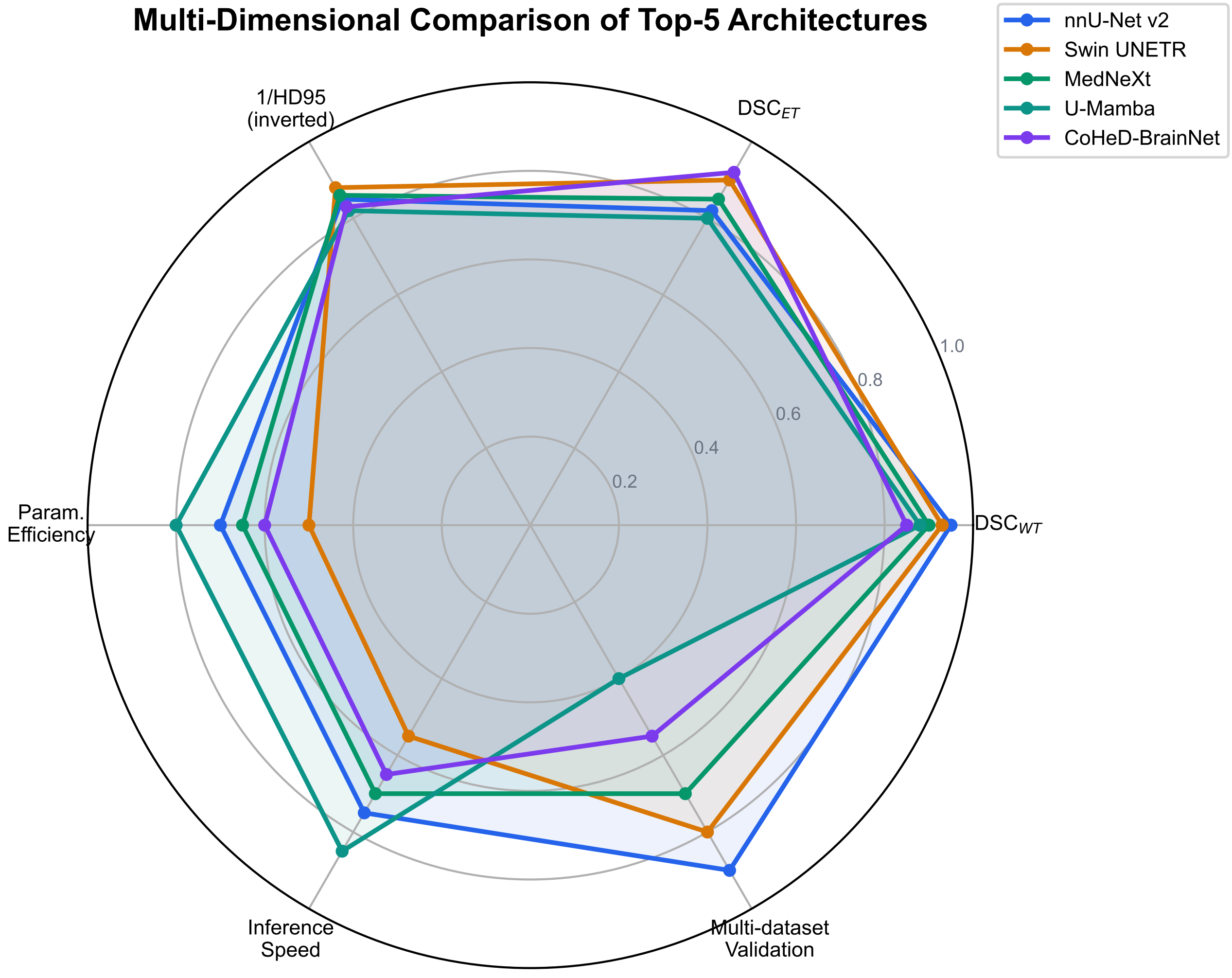

Supplement: Supplementary file 1 [file diagnostics-16-02806-s001.zip › Supplementary File S7_Figure_Data_and_Reproducibility.zip!/rendered_figures/radar_comparison.pdf]
