# Supplementary material for: Brain Tumor Segmentation and Grading on MRI Using Deep Learning: A Systematic Literature Review and Benchmark-Driven Comparative Analysis
Source: Diagnostics (Basel). 2026 Aug 31;16(17):2806. doi: 10.3390/diagnostics16172806 (PMC13565208; doi:10.3390/diagnostics16172806)
Supplement: Supplementary file 1 [file diagnostics-16-02806-s001.zip › Supplementary File S7_Figure_Data_and_Reproducibility.zip!/rendered_figures/segmentation_performance.pdf]

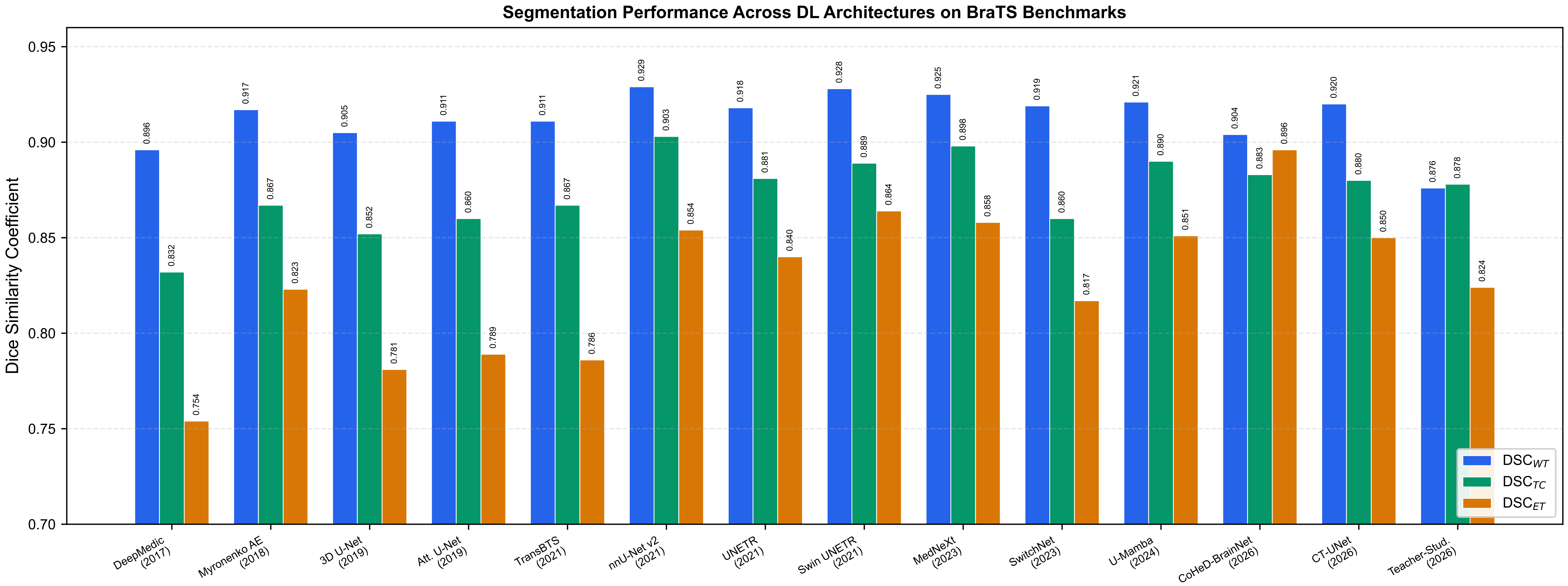

Segmentation Performance Across DL Architectures on BraTS Benchmarks
Dice Similarity Coefficient
0.70
0.75
0.80
0.85
0.90
0.95
DeepMedic (2017)
0.896
0.832
0.754
Myronenko AE (2018)
0.917
0.867
0.823
3D U-Net (2019)
0.905
0.852
0.781
Att. U-Net (2019)
0.911
0.860
0.789
TransBTS (2021)
0.911
0.867
0.786
nnU-Net v2 (2021)
0.929
0.903
0.854
UNETR (2021)
0.918
0.881
0.840
Swin UNETR (2021)
0.928
0.889
0.864
MedNeXt (2023)
0.925
0.898
0.858
SwitchNet (2023)
0.919
0.860
0.817
U-Mamba (2024)
0.921
0.890
0.851
CoHeD-BrainNet (2026)
0.904
0.883
0.896
CT-UNet (2026)
0.920
0.880
0.850
Teacher-Stud. (2026)
0.876
0.878
0.824
DSC_{WT}
DSC_{TC}
DSC_{ET}
