# Supplementary material for: Brain Tumor Segmentation and Grading on MRI Using Deep Learning: A Systematic Literature Review and Benchmark-Driven Comparative Analysis
Source: Diagnostics (Basel). 2026 Aug 31;16(17):2806. doi: 10.3390/diagnostics16172806 (PMC13565208; doi:10.3390/diagnostics16172806)
Supplement: Supplementary file 1 [file diagnostics-16-02806-s001.zip › Supplementary File S7_Figure_Data_and_Reproducibility.zip!/rendered_figures/taxonomy_diagram.pdf]

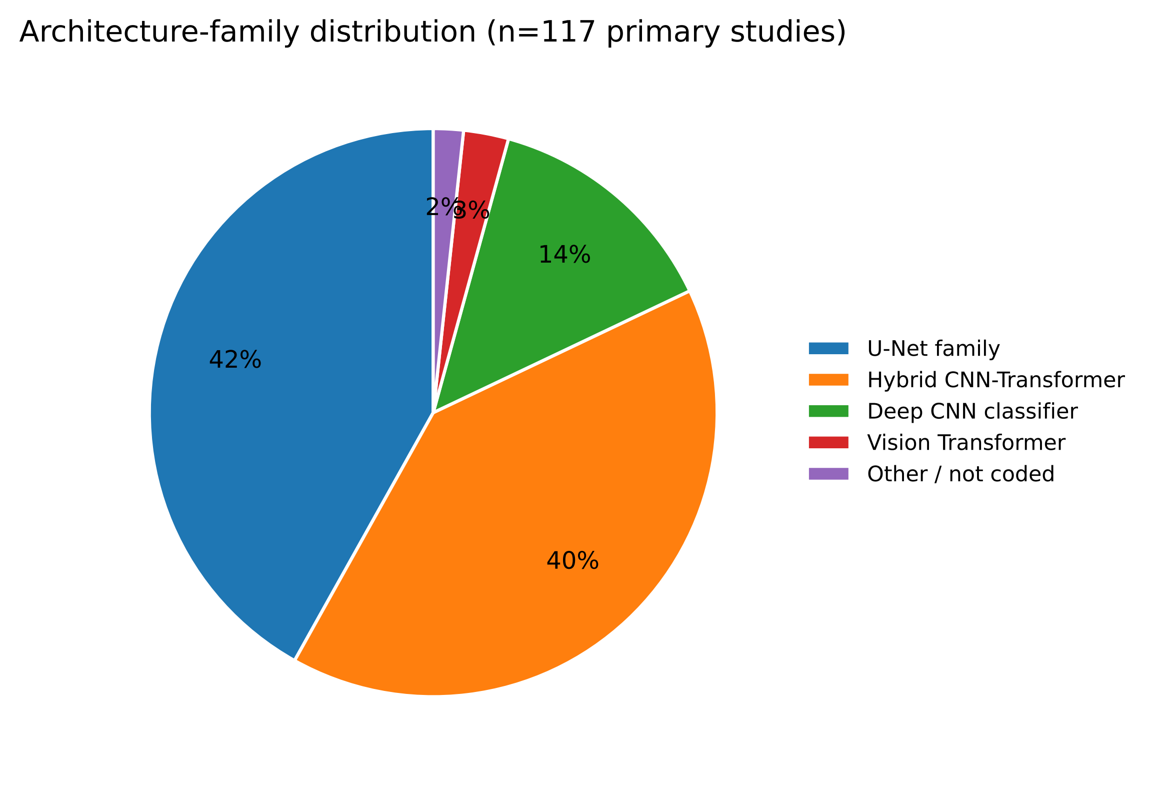
Architecture-family distribution (n=117 primary studies)
2%
3%
14%
42%
40%
U-Net family
Hybrid CNN-Transformer
Deep CNN classifier
Vision Transformer
Other / not coded
