# Supplementary material for: Brain Tumor Segmentation and Grading on MRI Using Deep Learning: A Systematic Literature Review and Benchmark-Driven Comparative Analysis
Source: Diagnostics (Basel). 2026 Aug 31;16(17):2806. doi: 10.3390/diagnostics16172806 (PMC13565208; doi:10.3390/diagnostics16172806)
Supplement: Supplementary file 1 [file diagnostics-16-02806-s001.zip › Supplementary File S7_Figure_Data_and_Reproducibility.zip!/rendered_figures/xai_adoption_trend.pdf]

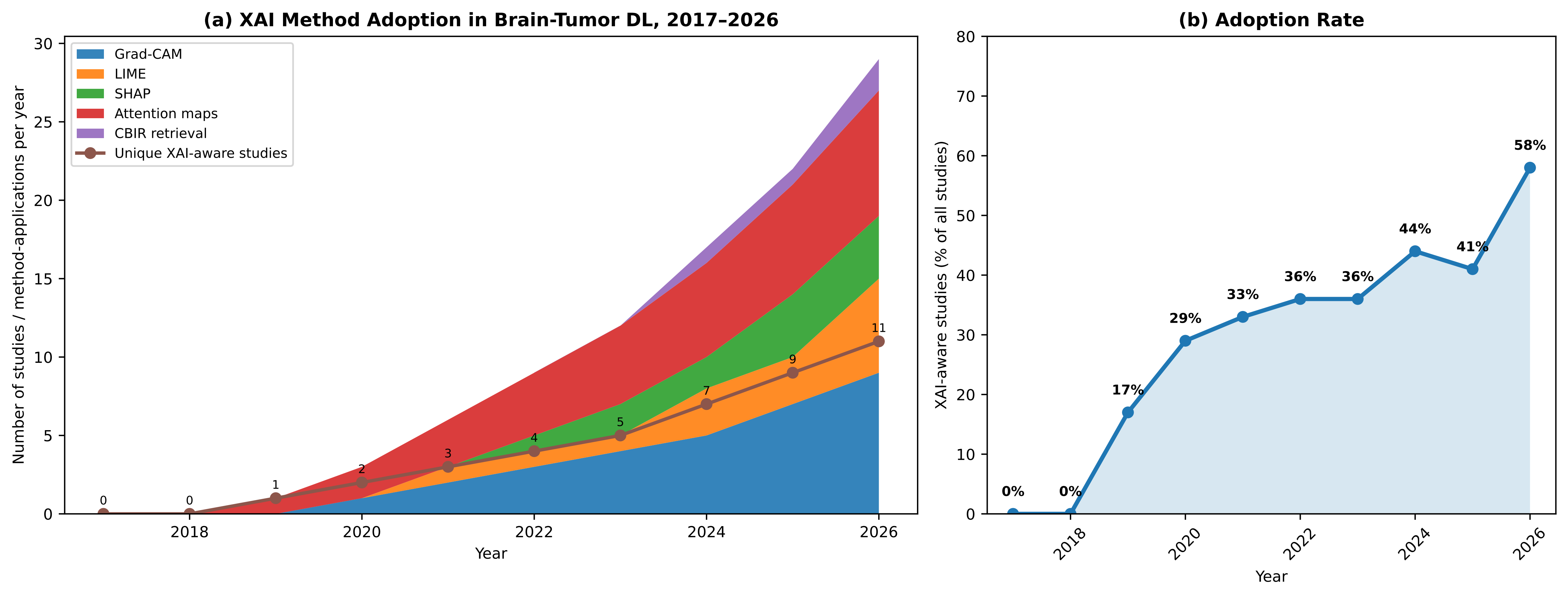

(a) XAI Method Adoption in Brain-Tumor DL, 2017–2026
Grad-CAM
LIME
SHAP
Attention maps
CBIR retrieval
Unique XAI-aware studies
Number of studies / method-applications per year
Year
0
0
1
2
3
4
5
7
9
11
(b) Adoption Rate
XAI-aware studies (% of all studies)
Year
0%
0%
17%
29%
33%
36%
36%
44%
41%
58%
