# Supplementary material for: Brain Tumor Segmentation and Grading on MRI Using Deep Learning: A Systematic Literature Review and Benchmark-Driven Comparative Analysis
Source: Diagnostics (Basel). 2026 Aug 31;16(17):2806. doi: 10.3390/diagnostics16172806 (PMC13565208; doi:10.3390/diagnostics16172806)
Supplement: Supplementary file 1 [file diagnostics-16-02806-s001.zip › Supplementary File S7_Figure_Data_and_Reproducibility.zip!/rendered_figures/year_distribution.pdf]

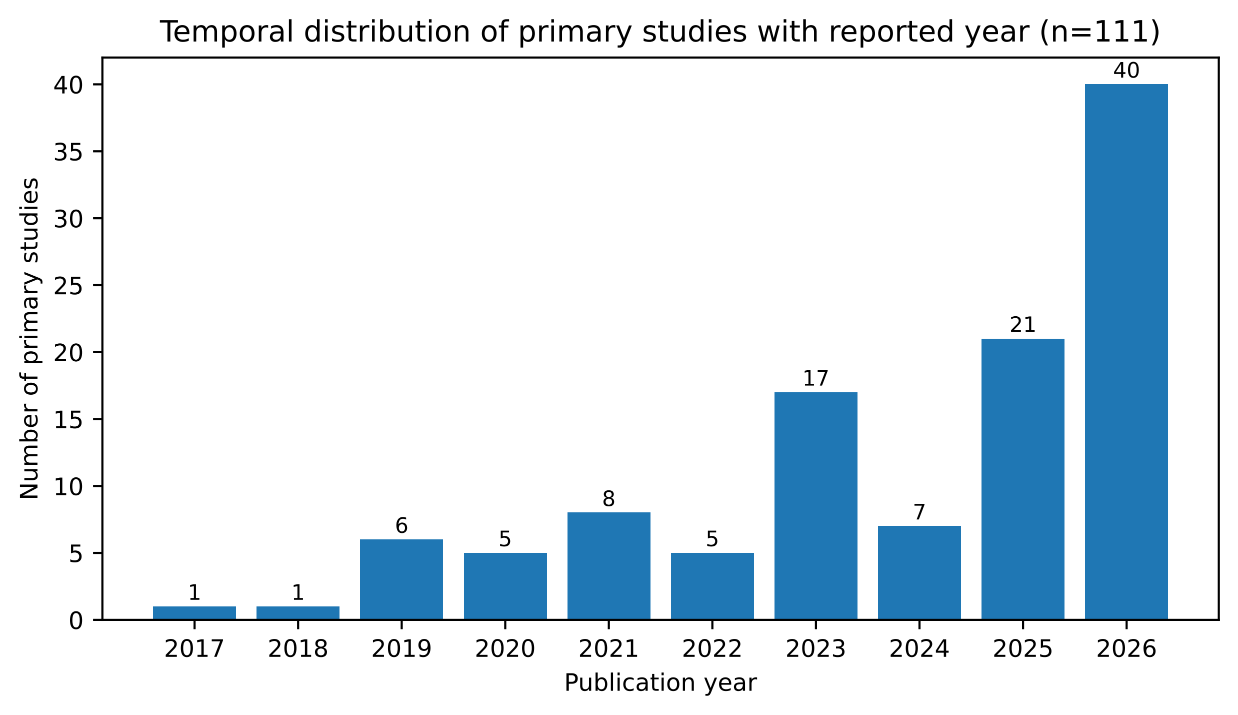
Temporal distribution of primary studies with reported year (n=111)
Number of primary studies
Publication year
0
5
10
15
20
25
30
35
40
2017
2018
2019
2020
2021
2022
2023
2024
2025
2026
1
1
6
5
8
5
17
7
21
40
